# Supplementary material for: Efficient Palladium-Catalyzed Synthesis of 2-Aryl Propionic Acids
Source: Molecules. 2020 Jul 28;25(15):3421. doi: 10.3390/molecules25153421 (PMC7435766; doi:10.3390/molecules25153421)
Supplement: Supplementary file 1 [file molecules-25-03421-s001.pdf]

Supporting Information

Efficient Palladium-Catalyzed Synthesis of 2-Aryl Propionic Acids

Helfried Neumann [1], Alexey G. Sergeev [2], Anke Spannenberg [1] and Matthias Beller [1,*]

[1] Leibniz-Institut für Katalyse e.V.; Albert-Einstein-Straße 29a 18059 Rostock, Germany; Helfried.Neumann@catalysis.de (H.N.); Anke.Spannenberg@catalysis.de (A.S.)

[2] University of Liverpool, Department of Chemistry, Liverpool L69 7ZD, United Kingdom; A.Sergeev@liverpool.ac.uk

* Correspondence: Matthias.Beller @catalysis.de

Experimental

Dioxane was purified by distillation from CaH_2. Unless otherwise noted, all reagents were used as received from commercial suppliers. The reaction was carried out in a 300 mL autoclave from the 4560 series of Parr Instruments®. Silica gel column chromatography was performed with 230–400 mesh ASTM silica gel from Merck. Mass spectra were obtained on an AMD 402/3 of AMD Intectra (EI, 70 eV). NMR data were recorded on a Bruker ARX 300 or on a Bruker 400. IR spectra of compounds were recorded using ATR method on a Nicolet Magna 550. GC analyses were performed on an HP 6890 equipped with a HP-5 capillary column (5% diphenylsiloxane 95% dimethylsiloxane, L=30 m, d=250 mm, dfilm=0.25 mm) and an FID detector. Gas chromatography-mass analysis was carried out on an Agilent HP-6890 instrument with an Agilent HP-5973 mass selective detector (EI) and HP-5 capillary column (polydimethylsiloxane with 5% phenyl groups, 30 m, 0.25 mm i.d., 0.25 μm film thickness) using helium carrier gas. The combustion analysis data for C/H/S were obtained from a C/H/N/S Mikroanalysator-TruSpec CHNS Micro (Leco). Quantitative GC analyses were referenced to internal hexadecane. All compounds were characterized by ^{1}H and ^{13}C NMR spectroscopy, IR, MS and elementary analysis.

Experimental procedure for the synthesis of 2-aryl-propionic acids

In a 100 mL Schlenk vial 0.75 mol% (11.79 mg) $Pd(OAc)_2$, 3.0 mol% (67.7 mg) NIPCDPP **9** were dissolved in 14 mL dioxane and 0.2 eq (410 μL) hexadecane as internal standard was added. Under argon 2.0 mL of this homogeneous yellow stock solution was transferred to each of six 4 mL vials equipped with a septum, needle and stirring bar, which are placed in an alloy plate. After 1 mmol of the corresponding aryl bromide and 1.5 eq (208 μL) of NEt_3 were added to each vial and a small sample was withdrawn for GC, the alloy plate was transferred into the 300 mL autoclave. The sealed autoclave was purged with ethylene several times and pressurized with 10 bar ethylene. Then, the reaction was run at 120 °C for 20 h. Afterwards the autoclave was cooled down to room temperature and the gas was carefully released. In the vials a grey precipitate were formed and the solution was still yellow. After a small sample for GC analyses was withdrawn, 83 μL of 6M HCL was added carefully. The reaction solution foams. Next, the autoclave was flushed with CO three times and the reaction was allowed to run at 40 bar CO. After 20 h, the autoclave was cooled down and the gas was released again. In order to determine the yield by GC, a sample of 100 μL of each reaction solution was esterified with (trimethylsilyl)diazomethane in the presence of 100 μL MeOH. The products were chromatographed after esterification in 10 mL MeOH and one drop cc H_2SO_4 (2 h refluxing) and characterized by NMR, elemental analysis and mass spectroscopy.

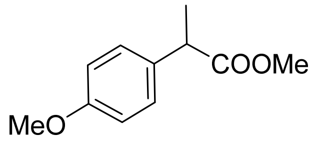

3a

Methyl 2-(4-(methoxy)phenyl)propanoate: Yield: 55%, Light yellow oil, R_f (EE/heptane= 0.25:10): 0.18; ^{1}H NMR (300 MHz, DMSO-d$_6$): δ = 7.18 (d, *J* = 8.5 Hz, 2H, CH), 6.87 (d, *J* = 8.5 Hz, 2H, CH), 3.71 (s, 3H, OCH_3), 3.71 (q, *J* = 7.1 Hz, 1H, C*H*CH_3), 3.55 (s, 3H, OCH_3), 1.34 (d, *J* = 7.1 Hz, 3H, CHC*H*$_3$); ^{13}C NMR (75 MHz, DMSO-d$_6$): δ = 174.5 (CO), 158.6 and 132.6 (2C), 128.4 and 113.9 (4CH), 55.0, 51.7 and 43.5 (2OCH_3, CHCH3), 18.6 (CH_3); MS (70 eV): m/z (%) = 194 (21) [M^+], 135 (100), 105 (9), 91 (8). IR (ATR) ν_{max} = 2952 (w), 1733 (vs), 1612 (m), 1511 (vs), 1456 (m), 1375 (w), 1334 (w), 1303 (w), 1244 (vs), 1206 (s), 1161 (s), 1116 (w), 1064 (m), 1033 (s), 968 (w), 858 (w), 833 (m), 814 (w), 788 (m), 732 cm $^{-1}$ (w); anal. calcd. (%) for $C_{11}H_{14}O_3$: C 68.02, H 7.27; found: C 68.18, H 7.30.

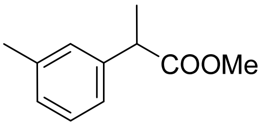

3b

Methyl 2-*m*-tolylpropanoate: Yield: 70%, light yellow oil; R_f (EE/heptane= 0.25:10): 0.26; ^{1}H NMR (300 MHz, $CDCl_3$): δ = 7.22 (m, 1H, CH), 7.09 (m, 3H, CH), 3.67 (s, 3H, OCH_3), 3.70 (q, *J* = 7.4 Hz, 1H, C*H*CH_3), 2.35 (s, 3H, *m*-CH_3), 1.49 (d, *J* = 7.4 Hz, 3H, CHC*H*$_3$); ^{13}C NMR (75 MHz, $CDCl_3$): δ = 175.0 (CO), 140.5 and 138.3 (2C), 128.5, 128.1, 127.9, 124.4 (4 CH), 52.0 and 45.3 (CHCH_3, OCH_3), 21.4 and 18.6 (2CH_3); MS (70 eV): m/z (%) = 178 (26) [M^+], 119 (100), 91 (14); IR (ATR) ν_{max} = 2980 (w), 2951 (w), 1733 vs), 1608 (w), 1455 (m), 1434 (m), 1375 (w), 1334 (m), 1236 (m), 1195 (s), 1168 s), 1156 (s), 1066 (m), 893 (w), 846 (w), 774 (m), 699 cm $^{-1}$ (s); anal. calcd. (%) for $C_{11}H_{14}O_2$: C 74.13, H 7.92; found: C 74.07, H 7.92.

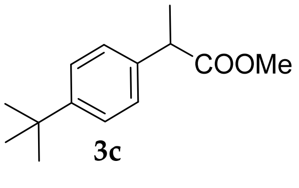

3c

Methyl 2-(4-*t*-butylphenyl)propanoate: Yield: 60%, light yellow oil; R_f (EE/heptane= 0.25:10): 0.31; ^{1}H NMR (300 MHz, DMSO-d$_6$): δ = 7.33 (d, *J* = 8.5 Hz, 2H, CH), 7.17 (d, *J* = 8.5 Hz, 2H, CH), 3.74 (q, *J* = 7.1 Hz, 1H, C*H*CH_3), 3.56 (s, 3H, OCH_3), 1.35 (d, *J* = 7.1 Hz, 3H, CHC*H*$_3$), 1.25 (s, 9H, *t*-Bu); ^{13}C NMR (75 MHz, DMSO-d$_6$): δ = 174.4 (CO), 149.3, 137.6 (2C), 127.0 and 125.3 (4 CH), 51.7 and 43.9 (CHCH_3, OCH_3), 34.1 (C), 31.1(*t*-Bu), 18.6 (CH_3); MS (70 eV): m/z (%) = 220 (18) [M^+], 205 (100), 161 (38), 145 (15), 131 (11); IR (ATR) ν_{max} = 2961 (w), 1736 (vs), 1509 (w), 1457 (w), 1434 (w), 1364 (w), 1335 (w), 1211 (m), 1162 (vs), 1111 (w), 1090 (w), 1063 (w), 1020 (w), 969 (w), 836 (m), 806 (w), 767 cm $^{-1}$ (w); anal. calcd. (%) for $C_{14}H_{20}O_2$: C 76.33, H 9.15; found: C 76.42, H 9.03.

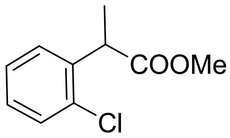

3d

Methyl 2-(2-chlorophenyl)propanoate: Yield: 21%, Light yellow oil, R_f (EE/heptane= 1:10): 0.30; ^{1}H NMR (300 MHz, $CDCl_3$): δ = 7.38 (dd, *J* = 1.7, 7.4 Hz, 1H, CH), 7.32 (dd, *J* = 2.2, 7.6 Hz, 1H, CH), 7.28 -7.16 ((m, 2H, 2CH), 4.22 (q, *J* = 7.2 Hz, 1H, C*H*CH_3), 3.69 (s, 3H, OCH_3), 1.50 (d, *J* = 7.2 Hz, 3H, CHCH_3); ^{13}C NMR (75 MHz, $CDCl_3$): δ = 174.7 (CO), 138.5 and 133.7 (2C), 129.7, 128.4 128.3 and 127.2 (4CH), 52.2 and 42.1 (OCH_3, *C*HCH3), 17.6 (CH_3); MS (70 eV): m/z (%) = 198 (2) [M^+], 164 (10), 163 (92), 141 (31), 139 (100), 103(55), 77(24). IR (ATR) ν_{max} = 2984(w), 2951 (w), 1735 (vs), 1477 (m), 1434 (m), 1376 (w), 1206 (s), 1169 (s), 1079 (m), 1036 (m), 956 (w), 860 (w), 807 (w), 751 (vs), 690 (m) cm^{-1}; anal. calcd. (%) for $C_{10}H_{11}ClO_2$: C 60.46, H 5.58; Cl 17.85 found: C 60.32, H 5.74, Cl 17.87.

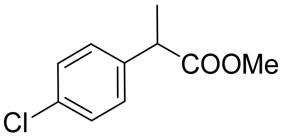

3e

Methyl 2-(4-chlorophenyl)propanoate: Yield: 36%, Light yellow oil, R_f (EE/heptane= 1:10): 0.30; ^{1}H NMR (300 MHz, DMSO-d_6): δ = 7.38 (d, *J* = 8.6 Hz, 2H, CH), 7.29 (d, *J* = 8.6 Hz, 2H, CH), 3.82 (q, *J* = 7.2 Hz, 1H, C*H*CH_3), 3.57 (s, 3H, OCH_3), 1.36 (d, *J* = 7.2 Hz, 3H, CHCH_3); ^{13}C NMR (75 MHz, DMSO-d_6): δ = 174.0 (CO), 139.6 and 131.6 (2C), 129.3 and 128.5 (4CH), 51.9 and 43.7 (OCH_3, *C*HCH3), 18.4 (CH_3); MS (70 eV): m/z (%) = 198 (22) [M^+], 139 (100), 103 (32), 77 (11). IR (ATR) ν_{max} = 2952 (w), 1734 (vs), 1492 (m), 1434 (w), 1410 (w), 1376 (w), 1332 (w), 1254 (w), 1206 (s), 1162 (s), 1091 (s), 1014 (m), 968 (w), 831 (m), 763 (m), 715 cm^{-1} (w); anal. calcd. (%) for $C_{10}H_{11}ClO_2$: C 60.46, H 5.58; found: C 60.54, H 5.77.

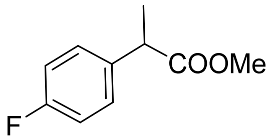

3f

Methyl 2-(4-fluorophenyl)propanoate: Yield: 54%, light yellow oil; R_f (EE/heptane= 0.25:10): 0.25; ^{1}H NMR (300 MHz, DMSO-d_6): δ = 7.31 (dd, *J* = 8.9, 5,6 Hz, 2H, CH), 7.14 (pt, *J* = 8.9 Hz, 2H, CH), 3.81 (q, *J* = 7.2 Hz, 1H, C*H*CH_3), 3.57 (s, 1H, OCH_3), 1.37 (d, *J* = 7.2 Hz, 3H, CHCH_3); ^{13}C NMR (75 MHz, DMSO-d_6): δ = 174.2 (CO), 161.2 (d, ^1J (C,F) = 242 Hz, C), 136.8 (d,^4J (C,F) = 3 Hz, C), 129.3 (d,^3J = (C,F) 8 Hz, CH), 115.3 (d,^2J (C,F) = 21 Hz, CH), 52.8 and 43.5 (*C*HCH_3, OCH_3), 18.6 (CH_3); MS (70 eV): m/z (%) = 182 (20) [M^+], 123 (100), 103 (28); IR (ATR) ν_{max} = 2982 (w), 2953 (w), 1734 (vs), 1603 (w), 1509 (vs), 1435 (m), 1377 (w), 1334 (w), 1221 (s), 1205 (s), 1157 (vs), 1056 (m), 1015 (w), 968 (w), 836 (s), 794 (m), 732 cm^{-1} (w); anal. calcd. (%) for $C_{10}H_{11}FO_2$: C 65.22, H 6.09; found: C 65.93, H 6.09.

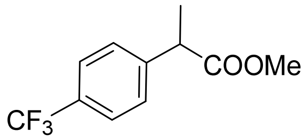

3g

Methyl 2-(4-(trifluoromethyl)phenyl)propanoate: Yield: 55%, Light yellow oil, R_f (EE/heptane= **0.2**5:10): 0.18; ^{1}H NMR (300 MHz, $CDCl_3$): δ = 7.58 (d, *J* = 8.4 Hz, 2H, CH), 7.42 (d, *J* = 8.4 Hz, 2H, CH), 3.79 (q, *J* = 7.2 Hz, 1H, C*H*CH_3), 3.67 (s, 3H, OCH_3), 1.52 (d, *J* = 7.2 Hz, 3H, CHC*H*$_3$); ^{13}C NMR (75 MHz, $CDCl_3$): δ = 174.2 (CO), 144.4 (C), 129.5 (q, ^2J = 32 Hz, C), 127.9, (CH), 125.6 (q, ^3J = 3.7 Hz, CH), 124.1 (q, ^1J = 276 Hz, CF_3), 52.2 and 45.3 ($CHCH_3$, OCH_3), 18.5 (CH_3); MS (70 eV): m/z (%) = 232 (22) [M^+], 173 (100), 153 (20), 133 (27). IR (ATR) ν_{max} = 2956 (w), 1737 (m), 1619 (w), 1436 (w), 1419 (w), 1323 (vs), 1210 (w), 1162 (s), 1115 (vs), 1065 (s), 1018 (m), 843 cm $^{-1}$ (m); anal. calcd. (%) for $C_{11}H_{11}F_3O_2$: C 56.90, H 4.77; found: C 56.44, H 4.68.

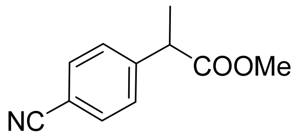

3h

Methyl 2-(4-cyanophenyl)propanoate: Yield: 54%, light yellow oil; R_f (EE/heptane= 1:7): 0.21; ^{1}H NMR (300 MHz, $CDCl_3$): δ = 7.62 (d, *J* = 8.5 Hz, 2H, CH), 7.41 (d, *J* = 8.5 Hz, 2H, CH), 3.78 (q, *J* = 7.1 Hz, 1H, C*H*CH_3), 3.67 (s, 3H, OCH_3), 1.51 (d, *J* = 7.1 Hz, 3H, CHC*H*$_3$); ^{13}C NMR (75 MHz, $CDCl_3$): δ = 173.8 (CO), 145.7, 118.7 and 111.3 (2C, CN), 132.4 and 128.4 (4 CH), 52.3 and 45.4 ($CHCH_3$, OCH_3), 18.3 (CH_3); MS (70 eV): m/z (%) = 189 (22) [M^+], 130 (100), 103 (19), 77 (7); IR (ATR) ν_{max} = 2984 (w), 2953 (w), 2229 (m, CN), 1733 (vs), 1608 (w), 1503 (w), 1455 (w), 1434 (w), 1209 (s), 1165 (s), 1068 (m), 1012 (w), 964 (w), 841 (m), 781 cm $^{-1}$ (w); anal. calcd. (%) for $C_{11}H_{11}NO_2$: C 69.83, H 5.86, N 7.40; found: C 69.99, H 5.96, N 7.34.

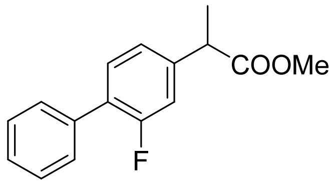

3i

Methyl 2-(2-fluorobiphenyl-4-yl)propanoate: Yield: 74%, light yellow oil; R_f (EE/heptane= 0.25:10): 0.21; ^{1}H NMR (400 MHz, $CDCl_3$): δ = 7.55 (m, 2H, CH), 7.48-7.34 (m, 4H, CH), 7.15 (m, 2H, CH), 3.77 (q, *J* = 7.2 Hz, 1H, C*H*CH_3), 3.71 (s, 1H, OCH_3), 1.55 (d, *J* = 7.2 Hz, 3H, CHC*H*$_3$); ^{13}C NMR (100 MHz, $CDCl_3$): δ = 174.4 (CO), 159.7 (d, ^1J (C,F) = 249 Hz, C), 141.8 (d,^3J (C,F) = 8 Hz, C), 135.5 (C), 127.8 (d,^2J = (C,F) 13 Hz, C), 130.8 (d, ^3J (C,F) = 4 Hz, CH), 128.9 (d, ^4J (C,F) = 3 Hz, CH), 128.4 (CH), 127 (CH), 123.5 (d, ^4J (C,F) = 3 Hz, CH), 115.2 (d, ^2J (C,F), = 24 Hz), 52.2 and 44.9 ($CHCH_3$, OCH_3), 18.4 (CH_3); MS (70 eV): m/z (%) = 258 (45) [M^+], 199 (100), 183 (13), 178 (16); IR (ATR) ν_{max} = 2982 (w), 2951 (w), 1734 (s), 1624 (w), 1582 (w), 1563 (w), 1516 (w), 1484 (m), 1450 (w), 1434 (w), 1417

(m), 1377 (w), 1332 (w), 1196 (m), 1169 (m), 1131 (m), 1071 (m), 1011 (w), 920 (m), 874 (w), 833(w), 766 (s), 724 (m), 697 cm $^{-1}$ (vs); anal. calcd. (%) for $C_{16}H_{15}FO_2$: C 74.40, H 5.85; found: C 74.23, H 6.09.

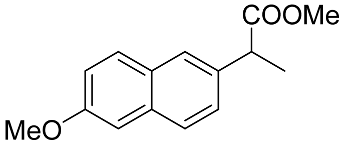

3j

Methyl 2-(6-methoxynaphthalen-2yl)propanoate: Yield: 36%, m.p.:59-60°C, light yellow solid, R_f (EE/heptane= 0.5:10): 0.15; ^{1}H NMR (300 MHz, $CDCl_3$): δ = 7.71 (d, *J* = 8.4 Hz, 2H, CH), 7.67 (d, *J* = 1.8 Hz, 1H, CH), 7.40 (dd, *J* = 8.4, 1.8 Hz, 1H, CH), 7.18-7.10 (m, 2H, CH), 3.91 (s, 3H, OCH_3), 3.67 (s, 3H, OCH_3), 3.87 (q, *J* = 7.2 Hz, 1H, C*H*CH_3), 1.59 (d, *J* = 7.2 Hz, 3H, CHC*H*$_3$); ^{13}C NMR (75 MHz, $CDCl_3$): δ = 175.1 (CO), 157.6, 135.6, 133.7 and 128.9 (4C), 129.2, 127.1, 126.1, 126.0, 119.0 and 105.5 (6CH), 55.3, 52.0, and 43.5 (2OCH_3, CHCH3), 18.6 (CH_3); MS (70 eV): m/z (%) = 244 (38) [M^+], 185 (100), 170 (13), 153 (8). IR (ATR) ν_{max} = 2976 (w), 1730 (s), 1604 (m), 1504 (w), 1484 (w), 1449 (m), 1392 (w), 1373 (w), 1331 (w), 1265 (m), 1199 (s), 1173 (s), 1092 (m), 1070 (m), 1028 (s), 968 (w), 957 (w), 923 (m), 896 (m), 856 (vs), 822 (s), 794 (m), 748 (w), 685 cm $^{-1}$ (m); anal. calcd. (%) for $C_{15}H_{16}O_3$: C 73.75, H 6.60; found: C 73.89, H 6.63.

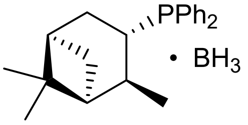

7-BH_3

Isopinocampheyl-diphenylphosphine-borane (ISPCDPP·BH_3).

A 250 mL three neck round bottomed flask equipped with a reflux condenser, stirring bar and septum was charged with 46.5 mmol (1.13 g) magnesium turnings and 12 mL absolute THF. To activate the magnesium turnings, 200 µL 1,2-dibromoethane was added and after the initiation of the Grignard reaction, 6.17 g (35.8 mmol) neoisopinocampheyl chloride in 14 mL THF was added dropwise to the solution to maintain the reaction. To complete the reaction, the mixture was heated to 50 °C and 30.2 mmol (0.84 eq, 5.43 mL) chlorodiphenylphosphine was added dropwise to the solution. After the mixture was refluxed for 2 h, a white precipitate was formed. The next day, 15 mL H_2O, 15 mL diethyl ether and 15 mL heptane were added to the solution. After separating the phases, the organic phase was washed with brine and dried over Na_2SO_4. The solvent was distilled off and after drying in vacuum, 7.77 g of crude product was achieved. For purification, the resulting phosphine was dissolved in 20 mL THF und 1.2 eq (28.76 mL) of a 1.0 M BH_3 THF solution was added. After stirring overnight, the solvent was again removed and the phosphine borane adduct was isolated by column chromatography on silica gel. The product was obtained a white solid (2.83g, yield 27%). Mp.: 154-155°C; R_f (EE/heptane= 0.25:10): 0.33; ^{1}H NMR (300 MHz, $CDCl_3$): δ = 7.91 (m, 2H, CH, arom.), 7.75 (m, 2H, CH, arom.), 7.54-7.45 (m, 3H, CH, arom.), 7.45-7.34 (m, 3H, CH, arom.), 2.83 (m, 1H, C*H*P), 2.50 (m, 1H, C*H*CH_3), 2.20 (m, 1H, CH_2), 2.13-1.92 (m, 2H, CH_2), 1.88

(m, 1H, CC*H*), 1.76 (m, 1H, CC*H*), 1.34 (d, *J* = 9.9 Hz, 1H, CH_2), 1.19 (s, 3H, CCH_3), 1.09 (s, 3H, CCH_3), 0.48 (d, *J* = 7.1 Hz, 3H, CHC*H*3); ^{13}C NMR (75 MHz, $CDCl_3$): δ = 133.1 (*J* = (Hz, CH), 132.9 (*J* = 8 Hz, CH), 131.2 (*J* = 3 Hz, CH), 131.1 (*J* = 3 Hz, CH), 130.6, 129.9 and 128.1 (C), 128.7 (*J* = 10 Hz, CH), 128.4 (*J* = 10 Hz CH), 47.8 (*J* = 7 Hz, CCH), 40.6 (*J* = 5 Hz, CCH), 39.2 (C), 35.9 (*J* = 3Hz, CHCH3), 30.7 (*J* = 32Hz, CHP), 30.4 (CH_2), 29.3 (*J* = 2.5 Hz, CH_2), 27.5 and 23.3 (CCH_3), 22.4 ($CHCH_3$); ^{31}P NMR (121 MHz, $CDCl_3$): δ = 25.8; MS (70 eV): m/z (%) = 322 (100) [M^+-BH_3], 307 (32), 279 (16), 253 (14), 186 (86), 183 (75), 108 (44), 93 (12), 81 (26); IR (ATR) ν_{max} = 2989 (w), 2904 (w), 2381 (m), 1435 (m), 1384 (w), 1142 (w), 1105 (m), 1064 (m), 747 (m), 730 (s), 691 cm $^{-1}$(vs); anal. calcd. (%) for $C_{22}H_{30}BP$: C 78.58, H 8.99, P 9.21; found: C 78.73, H 8.89, P 9.15.

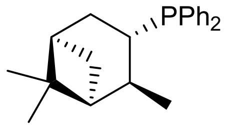

7

Isopinocampheyl-diphenylphosphine (ISPCDPP).

The procedure to release the free phosphine is analogous to **9** (see S10). The product was obtained as a white oil (860 mg, yield 90%) using 1 g borane adduct. R_f (EE/heptane= 1:10): 0.67; ^1H NMR ((300 MHz, $CDCl_3$, calibrated against grease with 0.08 ppm): δ = 8.33 (m, 2H, CH, arom.), 8.24 (m, 2H, CH, arom.), 8.10 (m, 3H, CH, arom.), 8.00 (m, 3H, CH, arom.), 3.17 (m, 1H, C*H*P), 3.08-2.08 (m, 3H, CH_2, C*H*CH3), 2.61 (m, 1H, CH, CH_2), 1.90 (s, 3H, CCH_3), 1.84 (d, *J* = 10.0 Hz,1H, CH_2), 1.81 (s, 3H, CCH_3), 1.21 (d, *J* = 7.2 Hz, 3H, CHC*H*3), ^{13}C NMR (75 MHz, $CDCl_3$): δ = 138.5, 138.3, 137.2, 137.0 (C), 134.1 (*J* = 19 Hz, CH), 133.8 (J = 19 Hz, CH), 128.8 (*J* = 12 Hz, CH), 128.3 (*J* = 7 Hz, CH), 128.1 (*J* = 7 Hz, CH), 48.3 (*J* = 6 Hz, CCH), 41.4 (*J* = 3 Hz, CCH), 39.7 (*J* = 19Hz, CHCH3), 39.2 (C), 32.3 (*J* = 17Hz, CH_2), 31.4 (*J* = 10Hz, CHP), 31,2 (CH_2), 27.6 (CCH_3), 23.2 (CCH_3), 22.1 (*J* = 2 Hz, $CHCH_3$); ^{31}P NMR (121 MHz, $CDCl_3$): δ = 5.53; MS (70 eV): m/z (%) = same mass spectrum as the borane adduct; IR (ATR) ν_{max} = 2898 (m), 1432 (m), 1261 (w), 1182 (w), 1092 (m),
1026 (w), 801 (w), 739 (m), 693 (s), 607 (w), 505 (m), 487 cm $^{-1}$(m); anal. calcd. (%) for $C_{22}H_{27}P$: C 81.95, H 8.44, P 9.61; found: C 81.19, H 8.44, P 9.79.

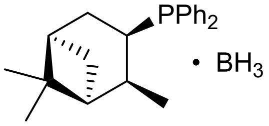

9-BH_3

Neoisopinocampheyl-diphenylphosphine-borane (NISPCDPP·BH_3).

In a 100 mL Schlenk tube 11 mmol (2.55g) isopinocampheyl methanesulfonate was dissolved in 15 mL absolute THF under argon atmosphere and cooled down to 0°C. After adding 17.6 mL of PPh_2K (0.5M in THF) the reaction mixture was stirred for 24 h at room temperature. The solution was quenched with 1 ml H_2O and the solvent was removed completely in vacuum. The residue was extracted with 20 mL ether and 10 mL H_2O and the aqueous phase was extracted three times with 15 ml diethyl ether. The combined organic layers were dried over Na_2SO_4. After removing the solvent, we obtained 2.8 g (80%) crude product. Next, we dissolved the product in 10 mL THF and added 1.2 eq (10.5 mmol) BH_3·THF to the solution. After the mixture was stirred overnight, the solvent was removed and the residue was purified by column chromatography over silica gel. The product was obtained as a white solid (881 mg, 30%). Mp.: 163-164°C; R_f (EE/heptane= 0.25:10): 0.22; ^1H NMR (300 MHz, $CDCl_3$): δ = 7.96 (m, 2H, arom.), 7.74 (m, 2H, arom.), 7.50 (m, 3H, arom.), 7.36

(m, 3H, arom.), 3.48 (psext, *J* = 9.9 Hz, 1H, CHP), 2.64 (m, 1H, C*H*CH_3), 2.42-2.18 (m, 2H, CH_2), 1.93 (m, 2H, 2CH), 1.45 (m, 2H, CH_2), 1.19 (s, 3H, CCH_3),1.13 (s, 3H, CCH_3), 1,04 (d, *J* = 7.7 Hz, 3H, CH_3); ^{13}C NMR (75 MHz, $CDCl_3$): δ = 132.3 (*J* = 6 Hz, CH, arom.), 132.2 (*J* = 6 Hz, CH, arom.), 131.6, 130.9, 130.1 and 129.4 (C), 131.1 (*J* = 3 Hz, CH, arom.), 130.5 (*J* = 3 Hz, CH, arom.), 128.9 (*J* = 10 Hz, CH, arom.), 128.4 (*J* = 10 Hz, CH, arom.), 48.8 (*J* = 5 Hz, CCH), 40.7 (*J* = 9 Hz, CCH), 39.6 (C), 36.5 (*C*HCH_3), 28.2 (CCH_3), 27.6 (*C*H_2CHCHP), 27.1 (*J* = 33 Hz, CHP), 27.0 (*J* = 4 Hz, CH_2), 22.9 (*C*CH_3) and 18.5 (CHCH_3); ^{31}P NMR (121 MHz, $CDCl_3$): δ = 17.5 , MS (70 eV): m/z (%) = 322 (42) [M^+-BH_3], 321 (51), 307 (50), 293 (37), 279 (27), 265 (75), 253 (11), 239 (12), 227 (25), 213 (74), 200 (11), 183 (100), 152 (12), 133 (12), 108 (37), 95 (10), 81 (23); IR (ATR) ν_{max} = 2895 (w), 2382 (w), 1462 (w), 1434 (m), 1386 (w), 1366 (w), 1190 (w), 1146 (w), 1104 (m), 1061 (s), 1029 (w), 746 (s), 735 (s), 690 (vs), 674 cm $^{-1}$ (s); anal. calcd. (%) for $C_{22}H_{30}BP$: C 78.58, H 8.99, P 9.21; found: C 78.71, H 8.93, P 9.15.

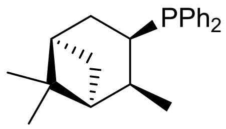

9

Neoisopinocampheyl-diphenylphosphine (NISPCDPP).

To release the free phosphine 500 mg neoisopinocampheyldiphenylphosphine-borane adduct was dissolved in 10 mL absolute morpholine and the mixture was stirred at 70°C for 2 h. After the mixture was cooled down, the morpholine was removed in oil pump vacuum and the residue was subjected to a column chromatography under argon atmosphere with degassed eluents. The product was obtained as colorless oil, which solidified upon standing to give a white solid (440 mg, yield: 98%). From this solid we were able to get crystals suitable for X-ray crystal structure analysis. R_f (EE/heptane= 1:10): 0.65; ^{1}H NMR (300 MHz, $CDCl_3$, referenced against the signals of silicon grease (◎◎0.08 ppm): δ = 7.60 (m, 2H, arom.), 7.48 (m, 2H, arom.), 7.34 (m, 3H, arom.), 7.26 (m, 3H, arom.), 3.20 (pquin, *J* = 9.6 Hz, 1H, CHP), 2.64 (m, 1H, C*H*CH_3), 2.21 (m, 1H, CH_2), 1.96 (m, 1H, CCH), 1.86 (m, 1H, CCH), 1.68 (m, 1H, CH_2), 1.46-1.42 (m, 3H, CH_2), 1.19 (s, 3H, CH_3),1.13 (s, 3H, CH_3), 1,13-1.08 (m, 3H, CHC*H*$_3$); ^{13}C NMR (75 MHz, $CDCl_3$): δ = 139.7 (*J* = 13 Hz, C), 138.7 (*J* = 13 Hz, C), 134.4 (*J* = 20 Hz, CH) 132.3 (*J* = 17 Hz, CH), 128.7, 128.3, 128.2, 128.1 and 128.0 (CH), 49.1 (*J* = 3 Hz, CCH), 36.7 (*J* = 11Hz, CCH), 39.2 (C), 36.7 (*J* = 11 Hz, *C*HCH_3), 30.6 (*J* = 17 Hz, CH_2), 28.1 (CH_2), 27.9 (CCH_3), 26.8 (*J* = 11Hz, CHP), 23.3 (*J* = 6Hz, CCH_3), 18.1 (*J* = 22 Hz, CHCH_3); ^{31}P NMR (121 MHz, $CDCl_3$): δ = -14.5; MS (70 eV): m/z (%) = same mass spectrum as the boran adduct; IR (ATR) ν_{max} = 2898 (m), 1433 (m), 1382 (m), 1260 (m), 1178 (m), 1095 (m), 799 (m), 735 (m), 691 (s), 561 (m), 538 (m), 511 (m), 472 cm $^{-1}$(m); anal. calcd. (%) for $C_{22}H_{27}P$: C 81.95, H 8.44, P 9.61; found: C 81.77, H 8.16, P 9.67.

X-ray crystal structure analysis of NISPCDPP (9)

Data were collected on a STOE IPDS II diffractometer using graphite-monochromatic Mo Kα radiation. The structure was solved by direct methods (SHELXS-97: Sheldrick, G. M. *Acta Cryst.* **2008**, *A64*, 112.) and refined by full-matrix least-squares procedures on F^2 (SHELXL-2018: Sheldrick, G. M. *Acta Cryst.* **2015**, *C71*, 3.). XP (Bruker AXS) was used for graphical representation.

Crystal data of **2c**: $C_{22}H_{27}P$, M = 322.40, orthorhombic, space group $P2_12_12_1$, a = 6.9541(3), b = 14.6577(6), c = 18.0759(7) Å, V = 1842.50(13) Å^3, T = 200(2) K, Z = 4, 28878 reflections measured, 4452 independent reflections (R_{int} = 0.047), final R values ($I > 2\sigma(I)$): R_1 = 0.0382, wR_2 = 0.0948, final R values (all data): R_1 = 0.0629, wR_2 = 0.1016, 187 parameters.

CCDC 2009413 contains the supplementary crystallographic data for this paper. These data are provided free of charge by the joint Cambridge Crystallographic Data Centre and Fachinformationszentrum Karlsruhe Access Structures service www.ccdc.cam.ac.uk/structures.

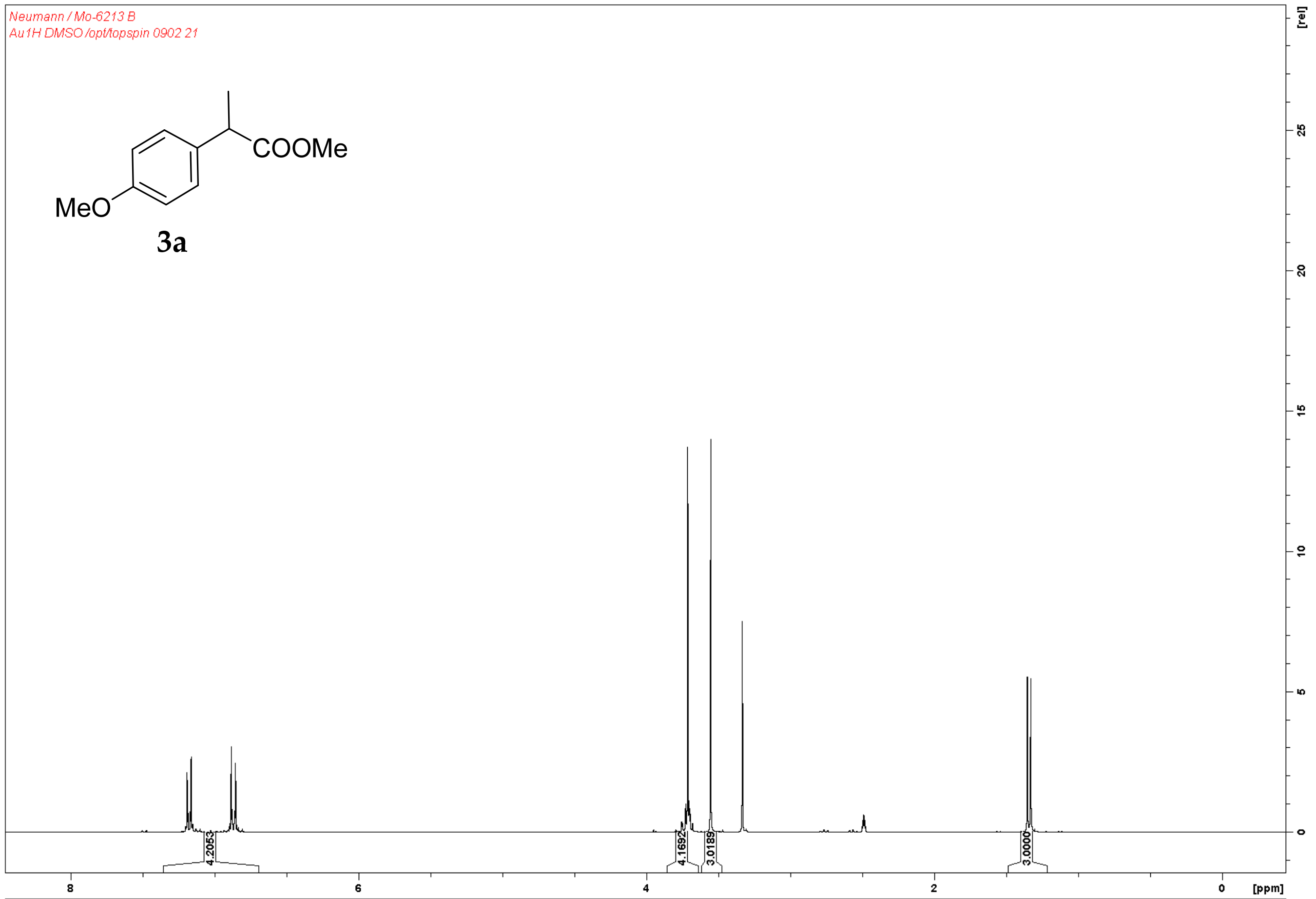
Neumann / Mo-6213 B
Au1H DMSO /opt/topspin 0902 21
COOMe
MeO
3a
4.2053
4.1692
3.0188
3.0000
[ppm]
[rel]

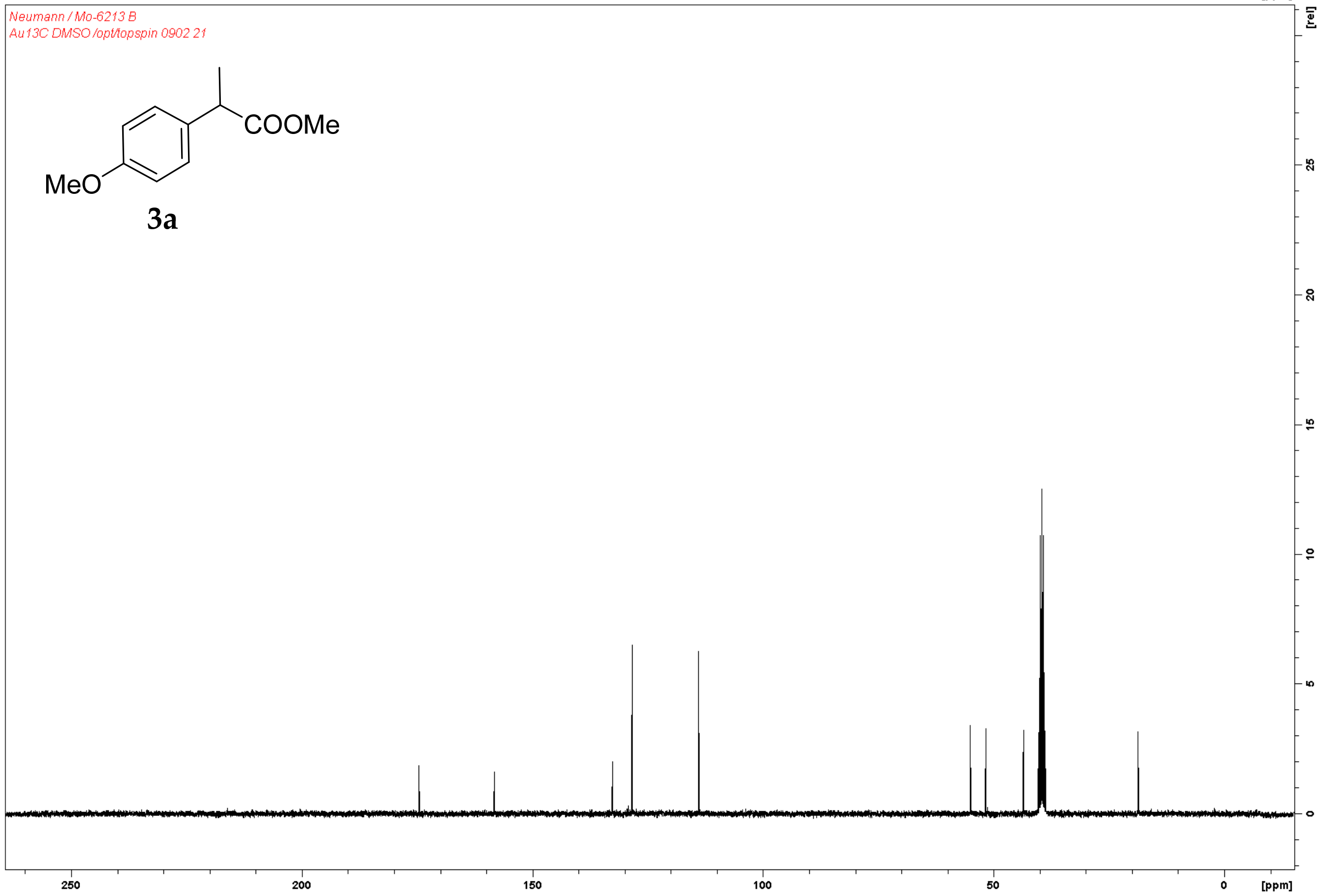
Neumann / Mo-6213 B
Au13C DMSO /opt/topspin 0902 21
COOMe
MeO
3a
[ppm]
[rel]

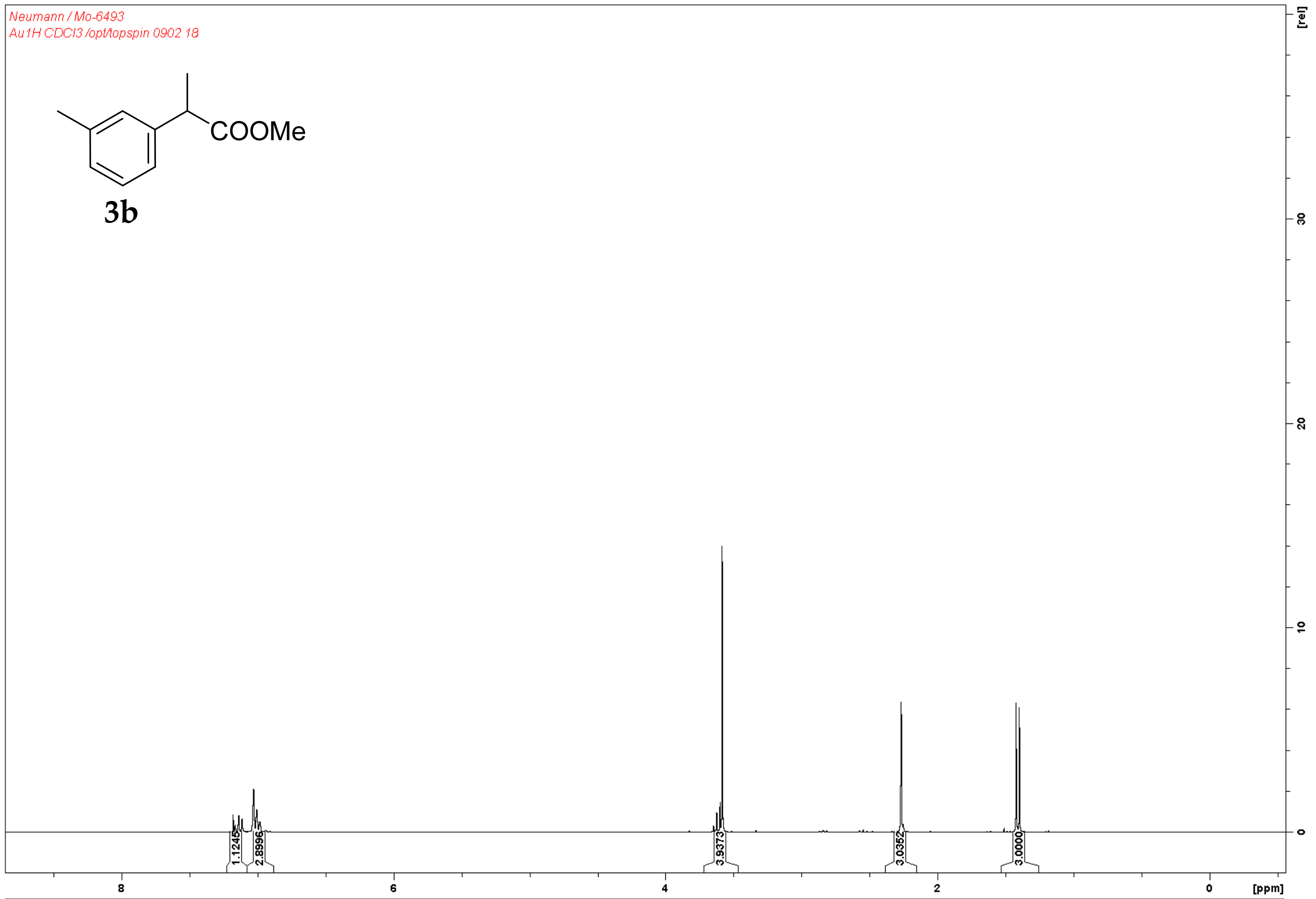
Neumann / Mo-6493
Au1H CDCl3 /opt/topspin 0902 18
COOMe
3b
1.1245
2.8996
3.9373
3.0352
3.0000
[ppm]
[rel]

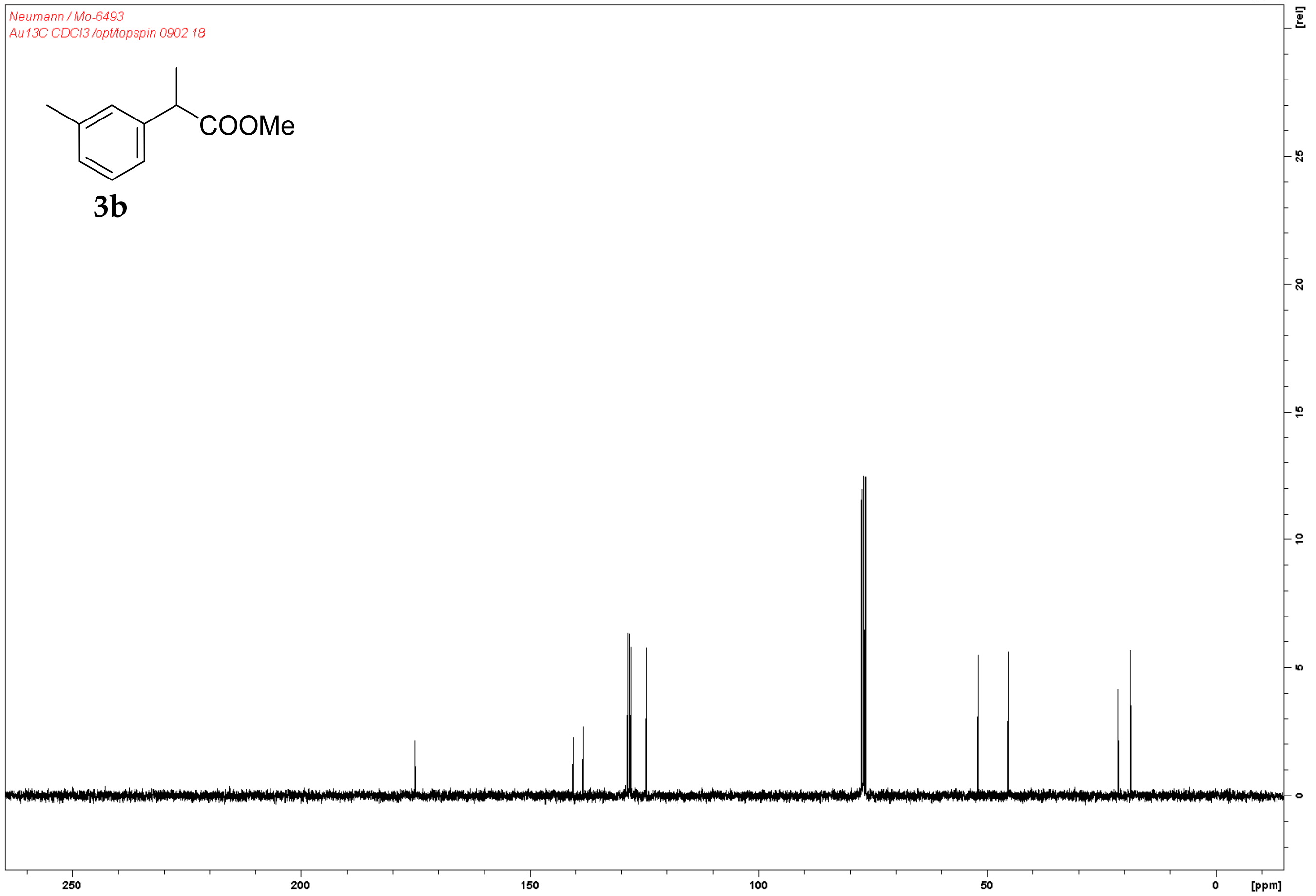
Neumann / Mo-6493
Au13C CDCl3 /opt/topspin 0902 18
COOMe
3b
[ppm]
[rel]

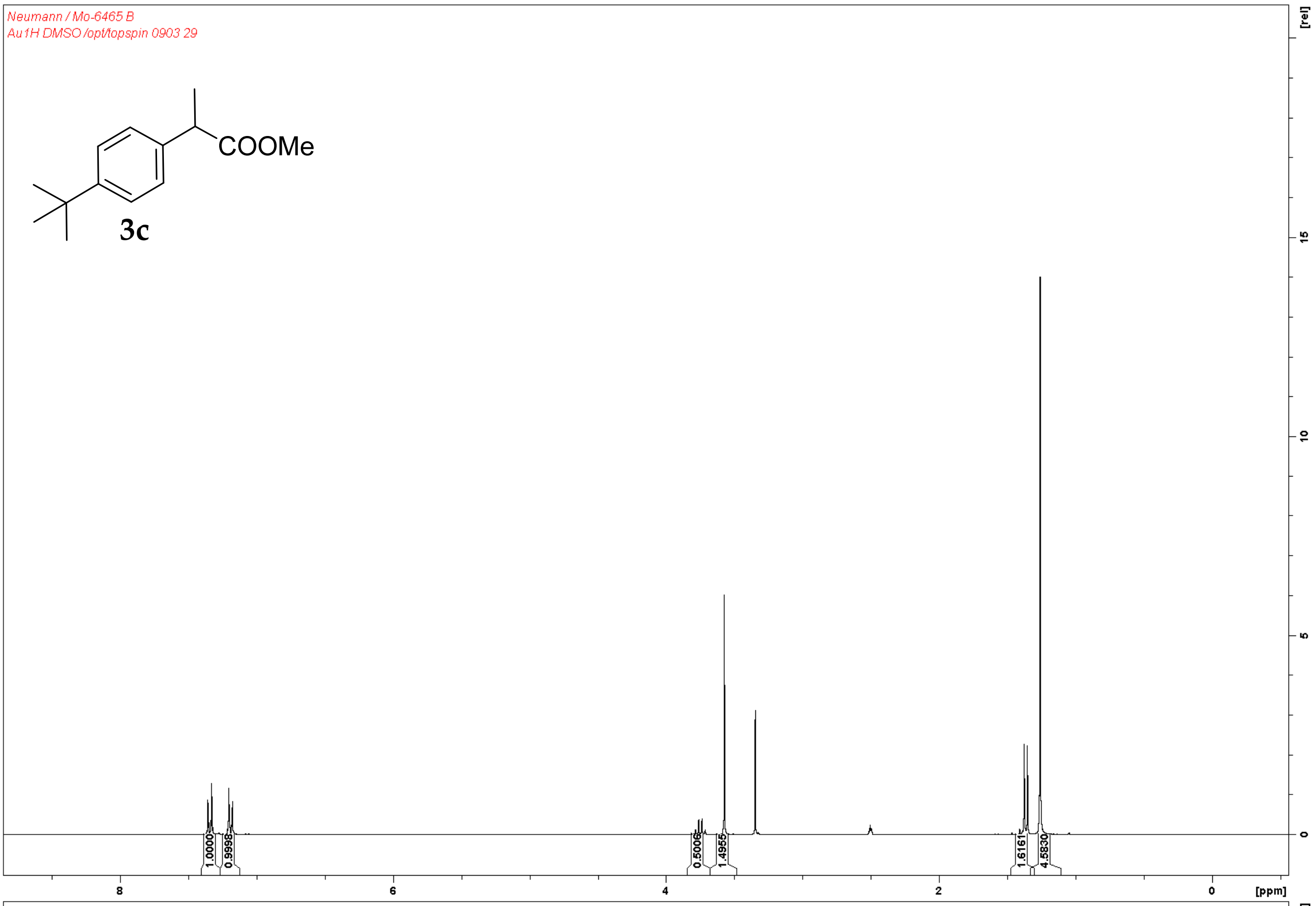
Neumann / Mo-6465 B
Au1H DMSO /opt/topspin 0903 29
COOMe
3c
1.0000
0.9998
0.5006
1.4955
1.6161
4.5830
[ppm]
[rel]

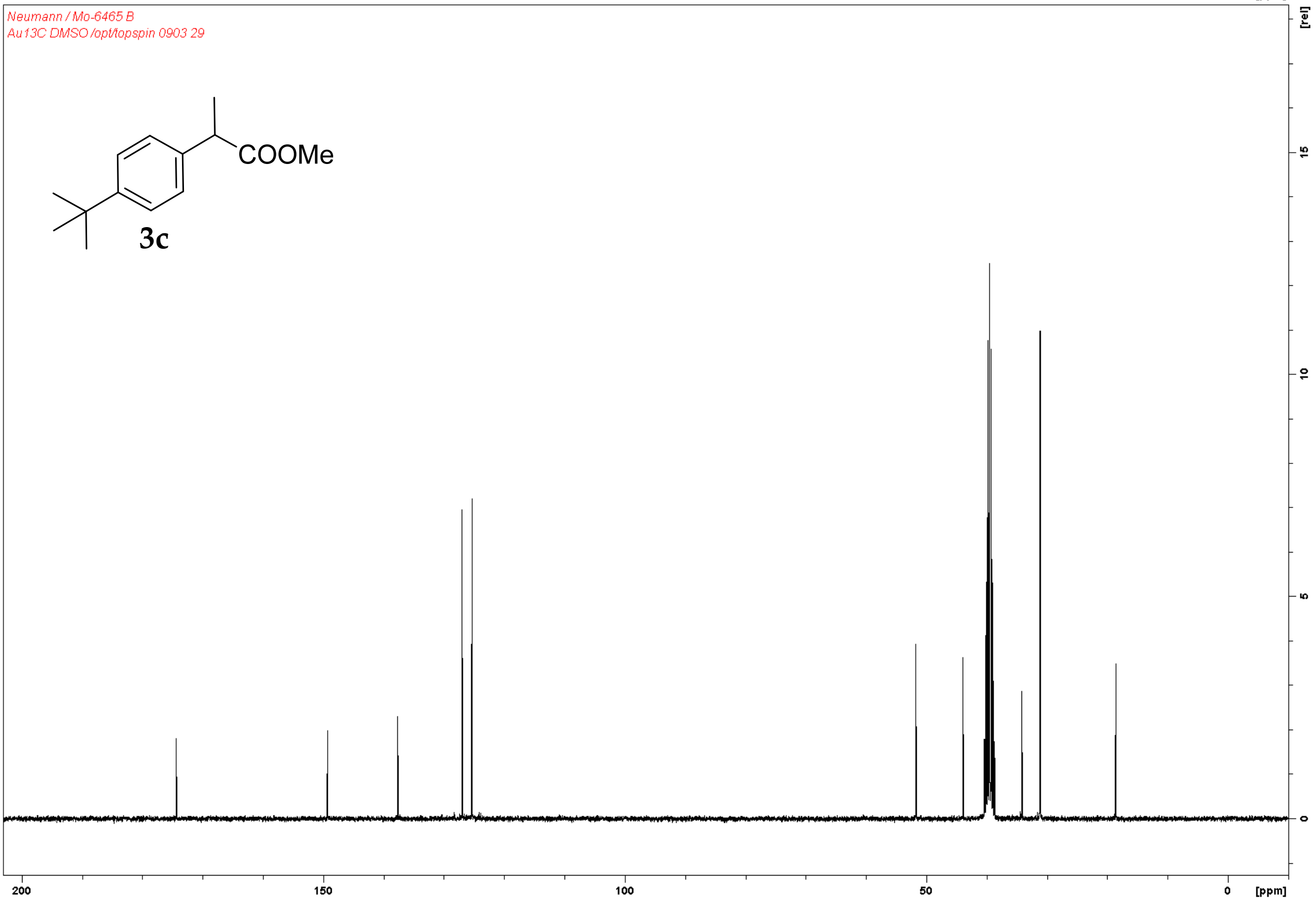
Neumann / Mo-6465 B
Au13C DMSO /opt/topspin 0903 29
COOMe
3c
[ppm]
[rel]

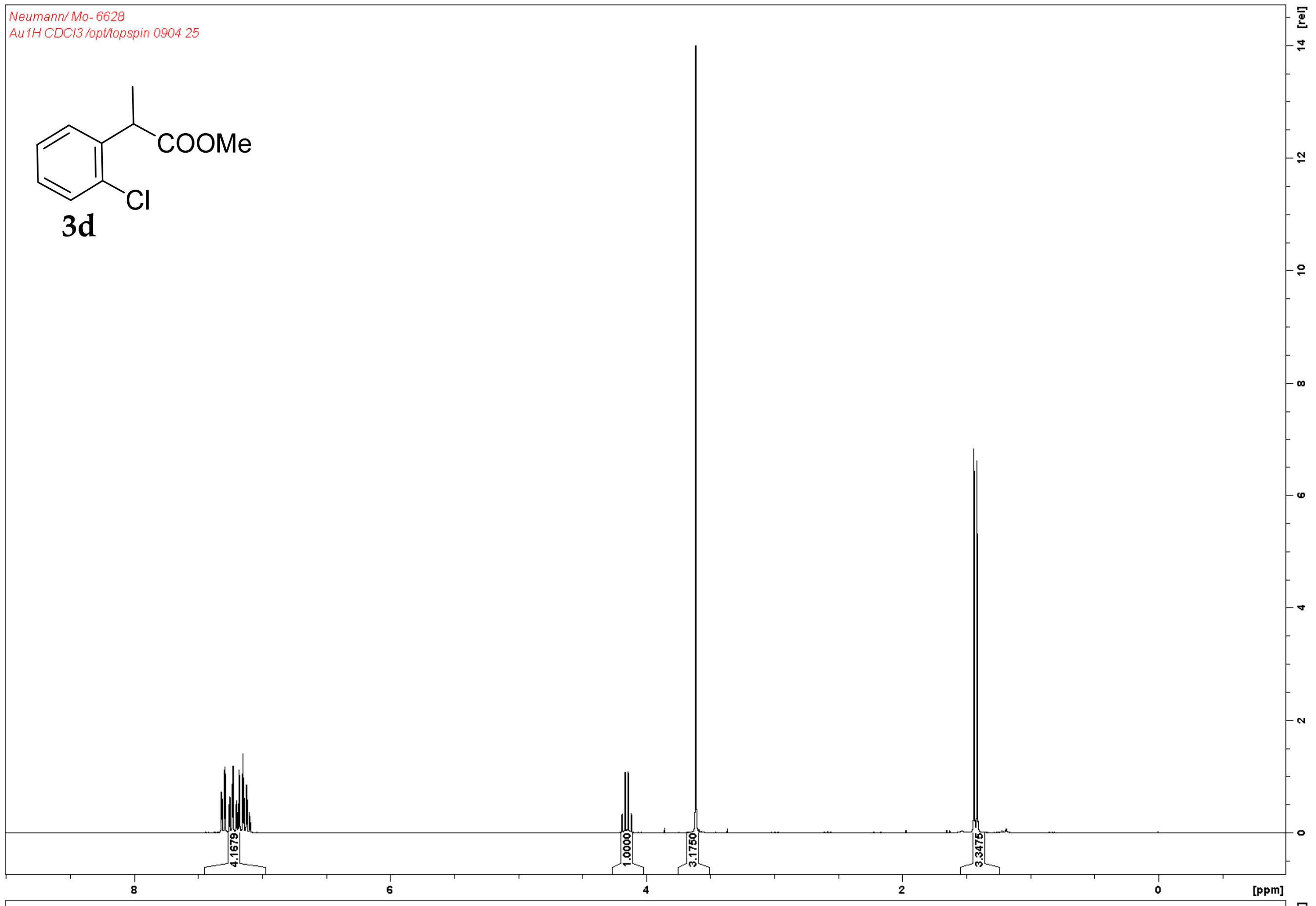
Neumann/ Mo- 6628
Au1H CDCl3 /opt/topspin 0904 25
COOMe
Cl
3d
4.1679
1.0000
3.1750
3.3475
[ppm]
[rel]

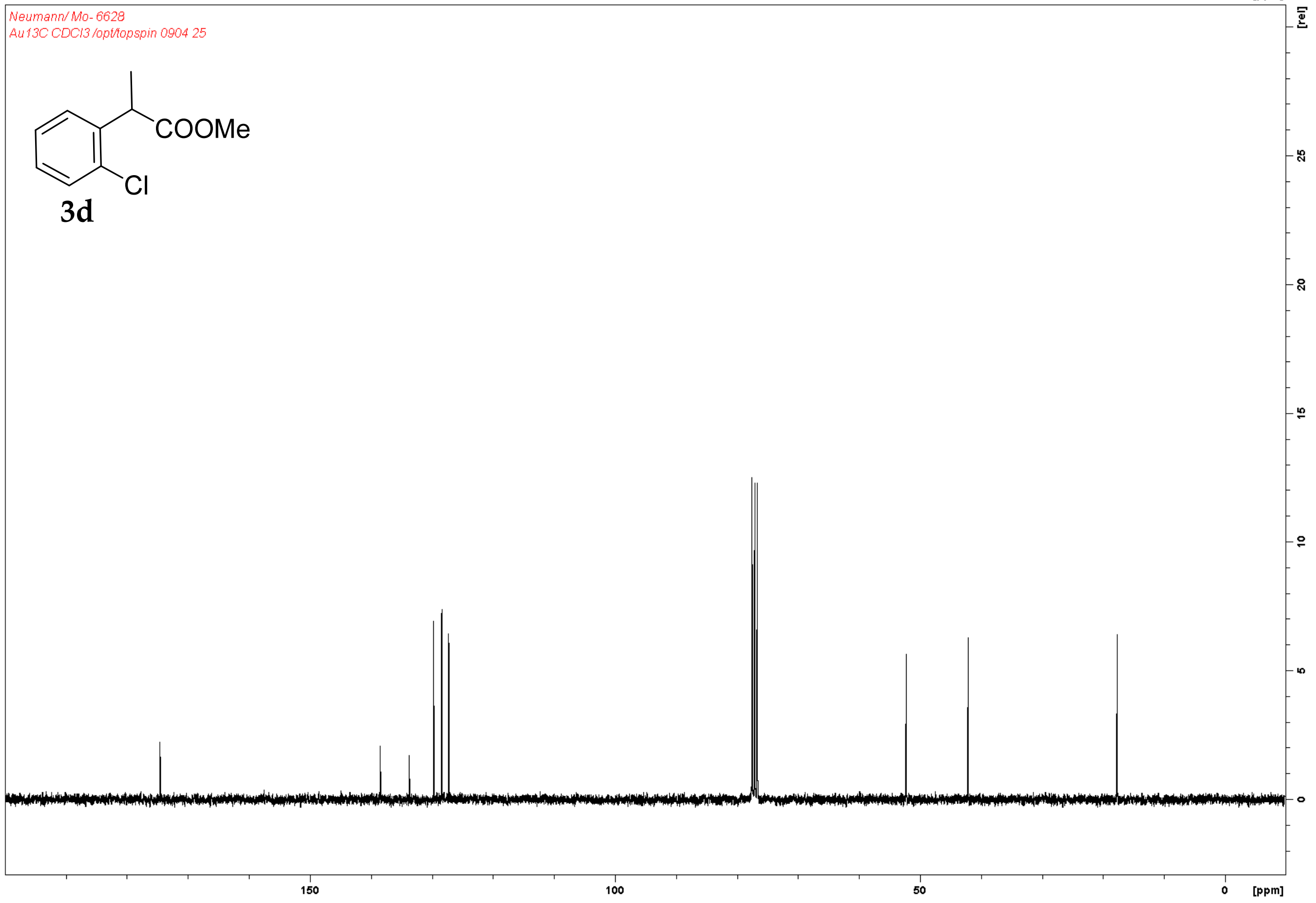
Neumann/ Mo- 6628
Au13C CDCl3 /opt/topspin 0904 25
COOMe
Cl
3d
[ppm]
[rel]

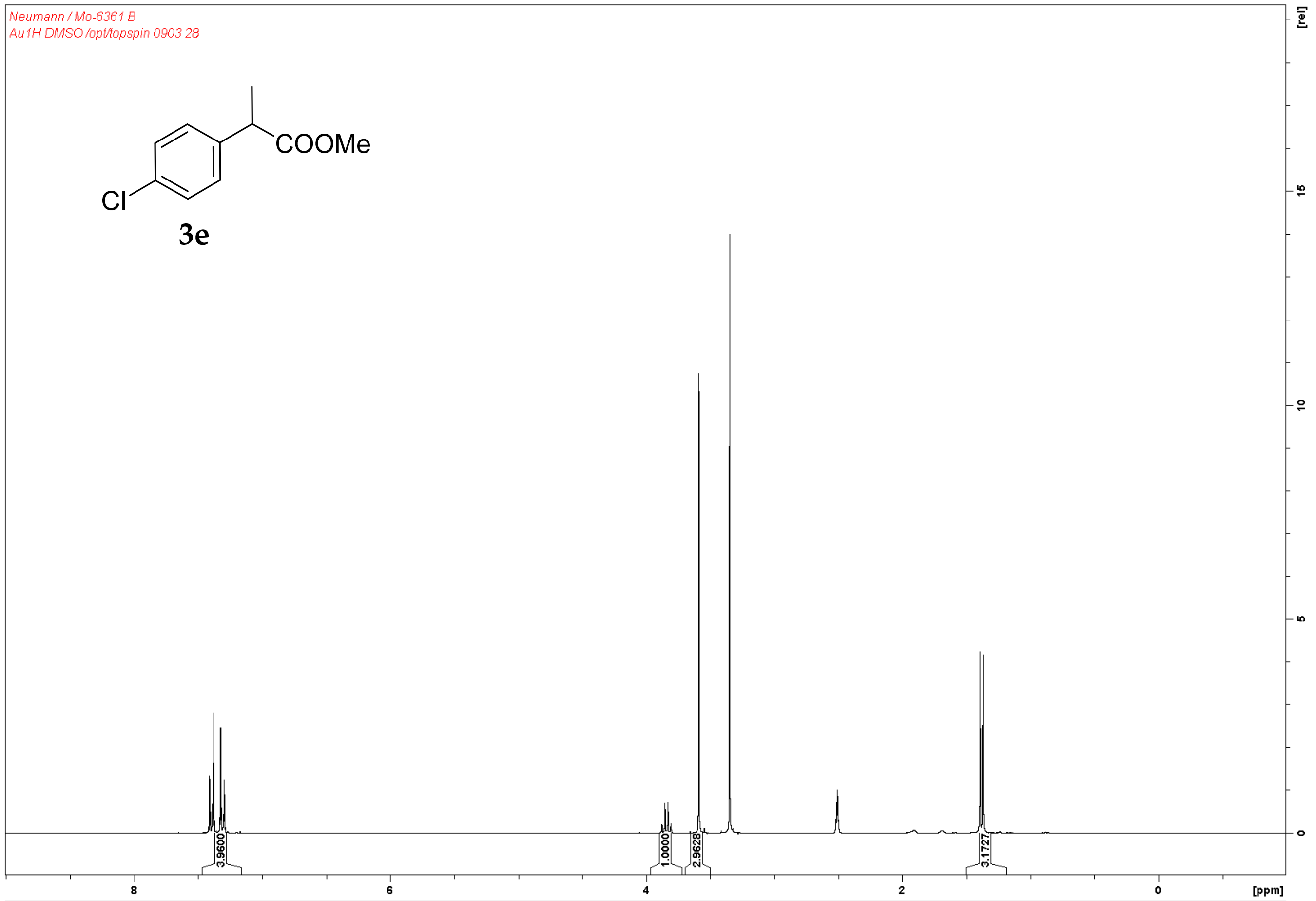
Neumann / Mo-6361 B
Au1H DMSO /opt/topspin 0903 28
COOMe
Cl
3e
3.9600
1.0000
2.9628
3.1727
[ppm]
[rel]

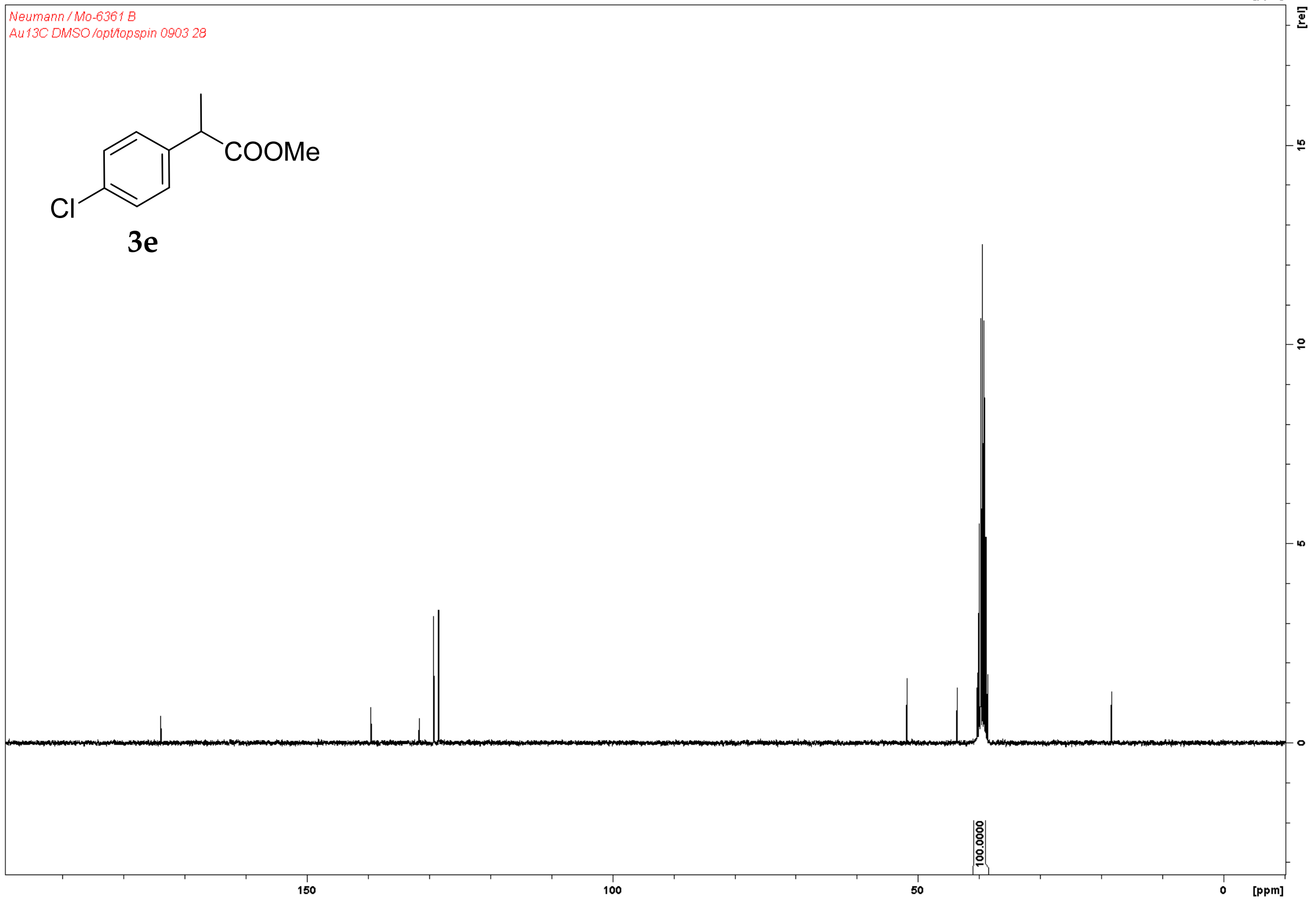
Neumann / Mo-6361 B
Au13C DMSO /opt/topspin 0903 28
COOMe
Cl
3e
100.0000
[ppm]
[rel]

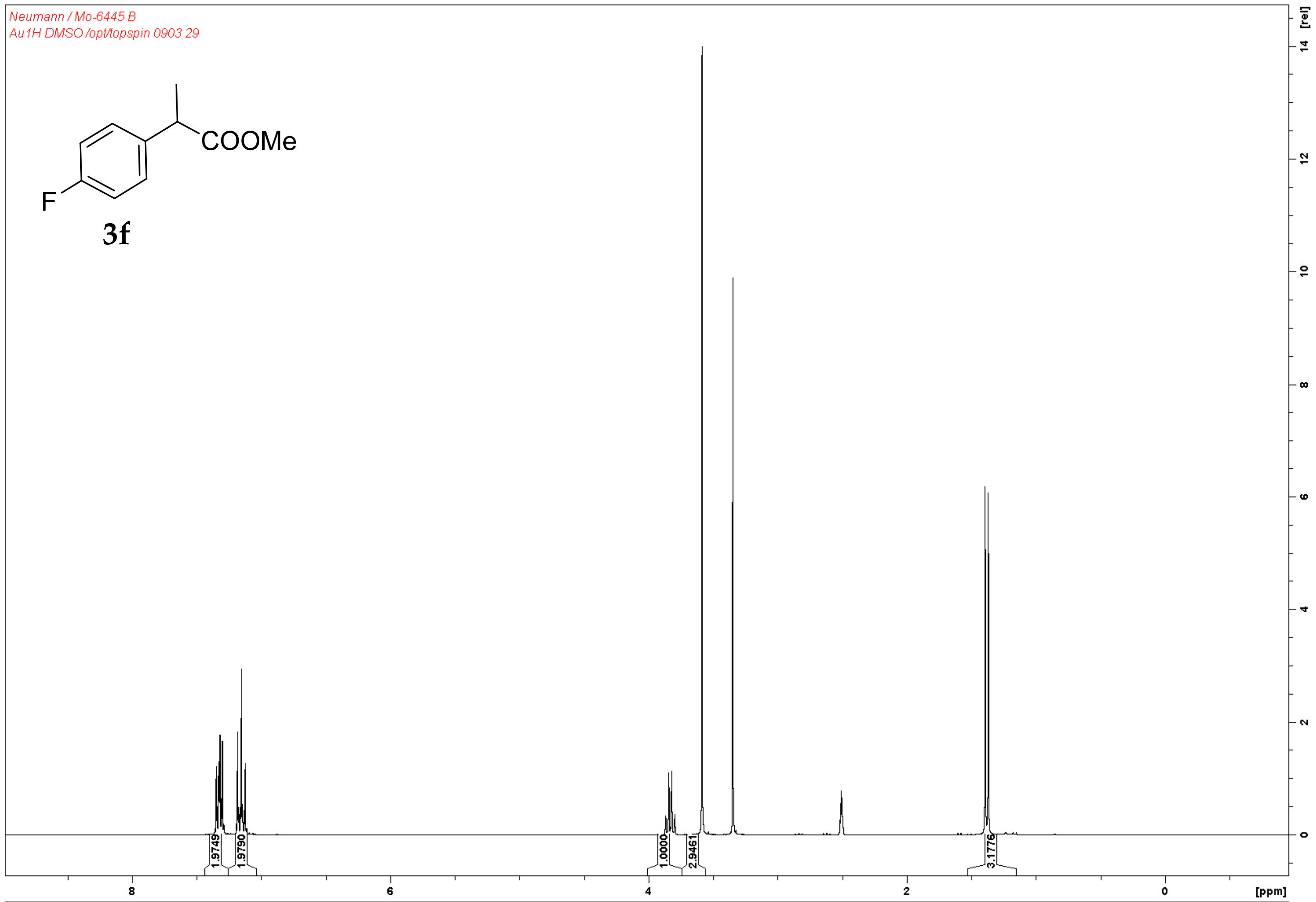

Neumann / Mo-6445 B
Au1H DMSO /opt/topspin 0903 29
COOMe
F
3f
1.9749
1.9790
1.0000
2.9461
3.1776
[rel]
[ppm]

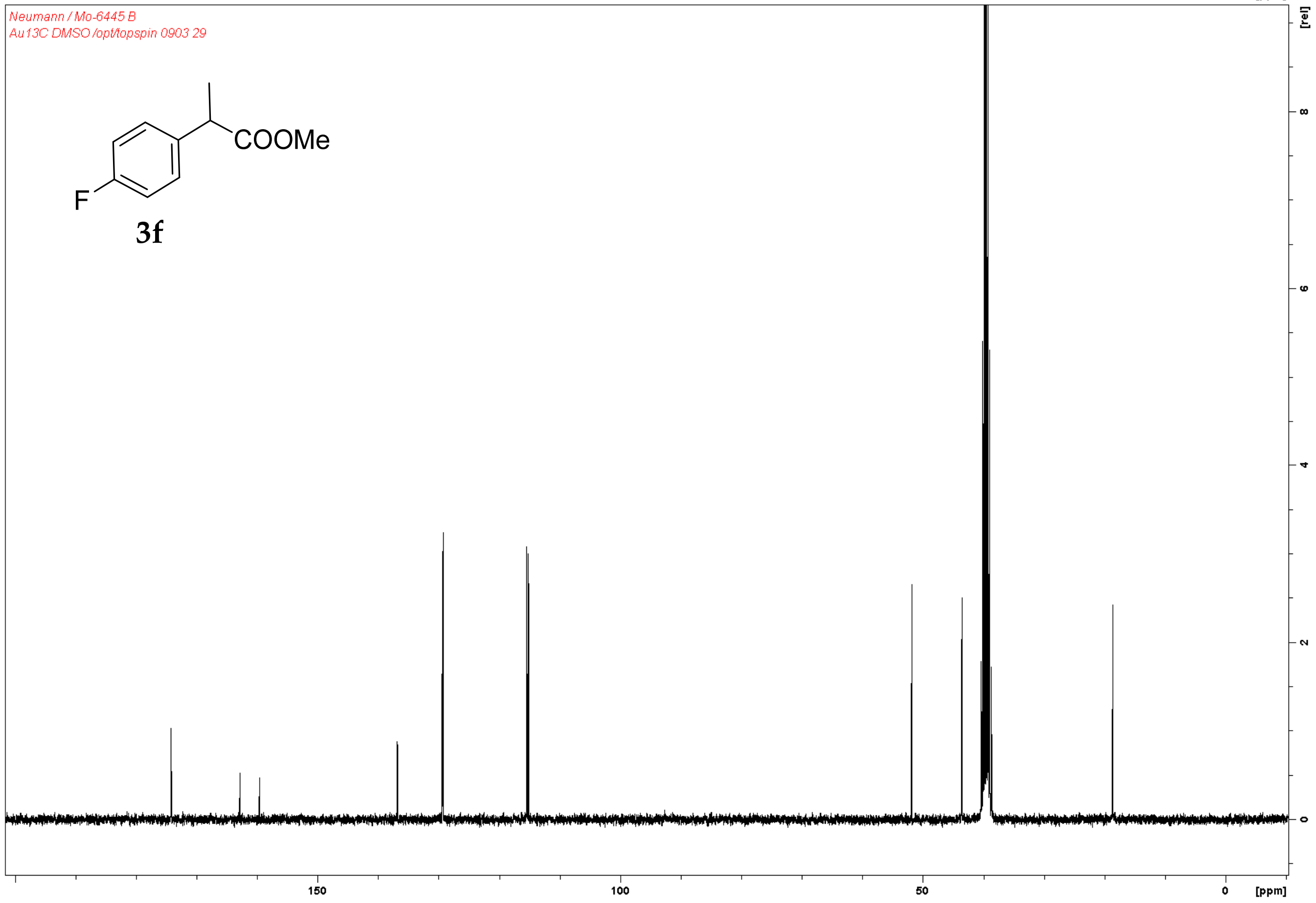

Neumann / Mo-6445 B
Au13C DMSO /opt/topspin 0903 29
COOMe
F
3f
[rel]
[ppm]

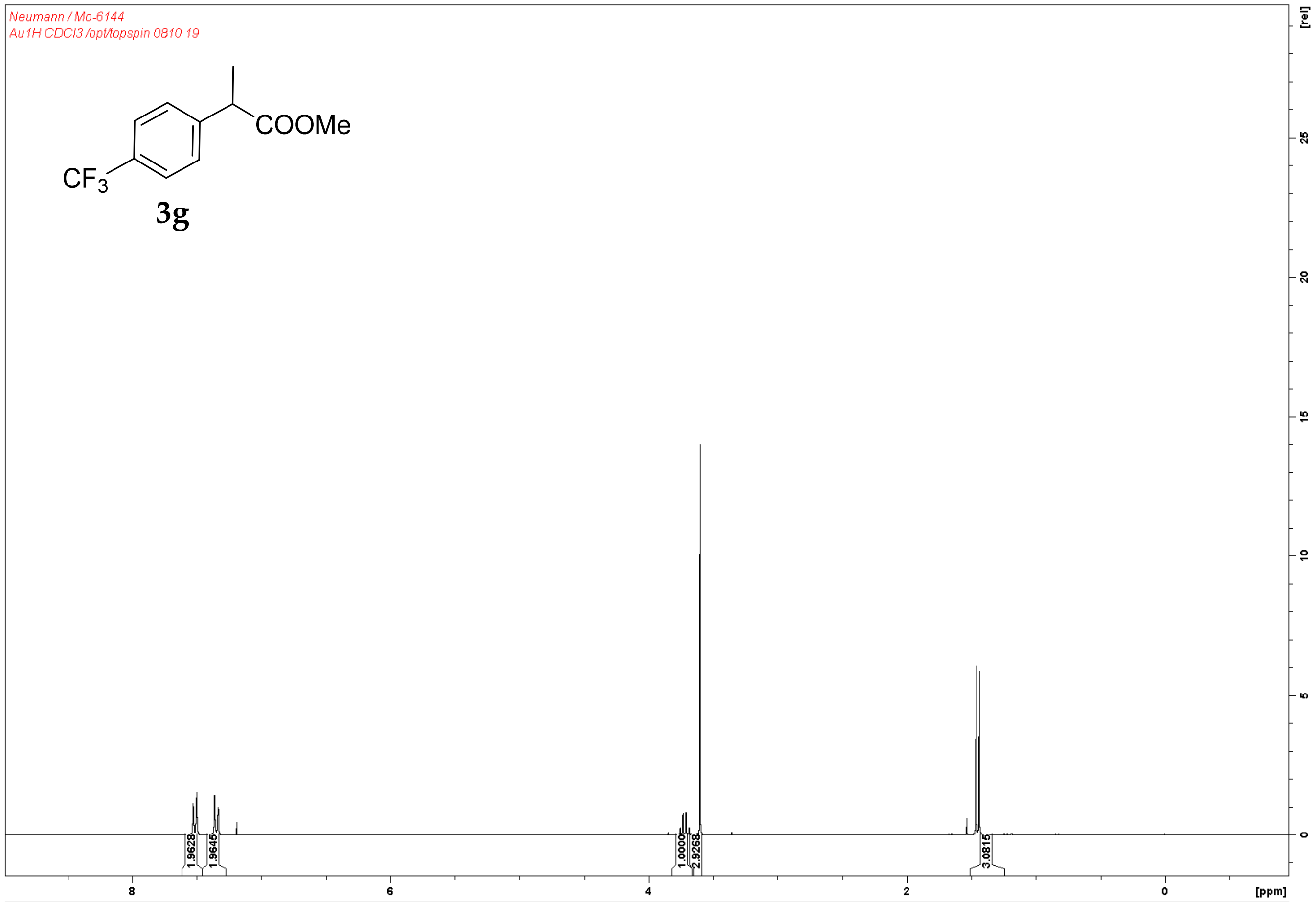
Neumann / Mo-6144
Au1H CDCl3 /opt/topspin 0810 19
COOMe
CF3
3g
1.9628
1.9645
1.0000
2.9268
3.0815
[rel]
[ppm]

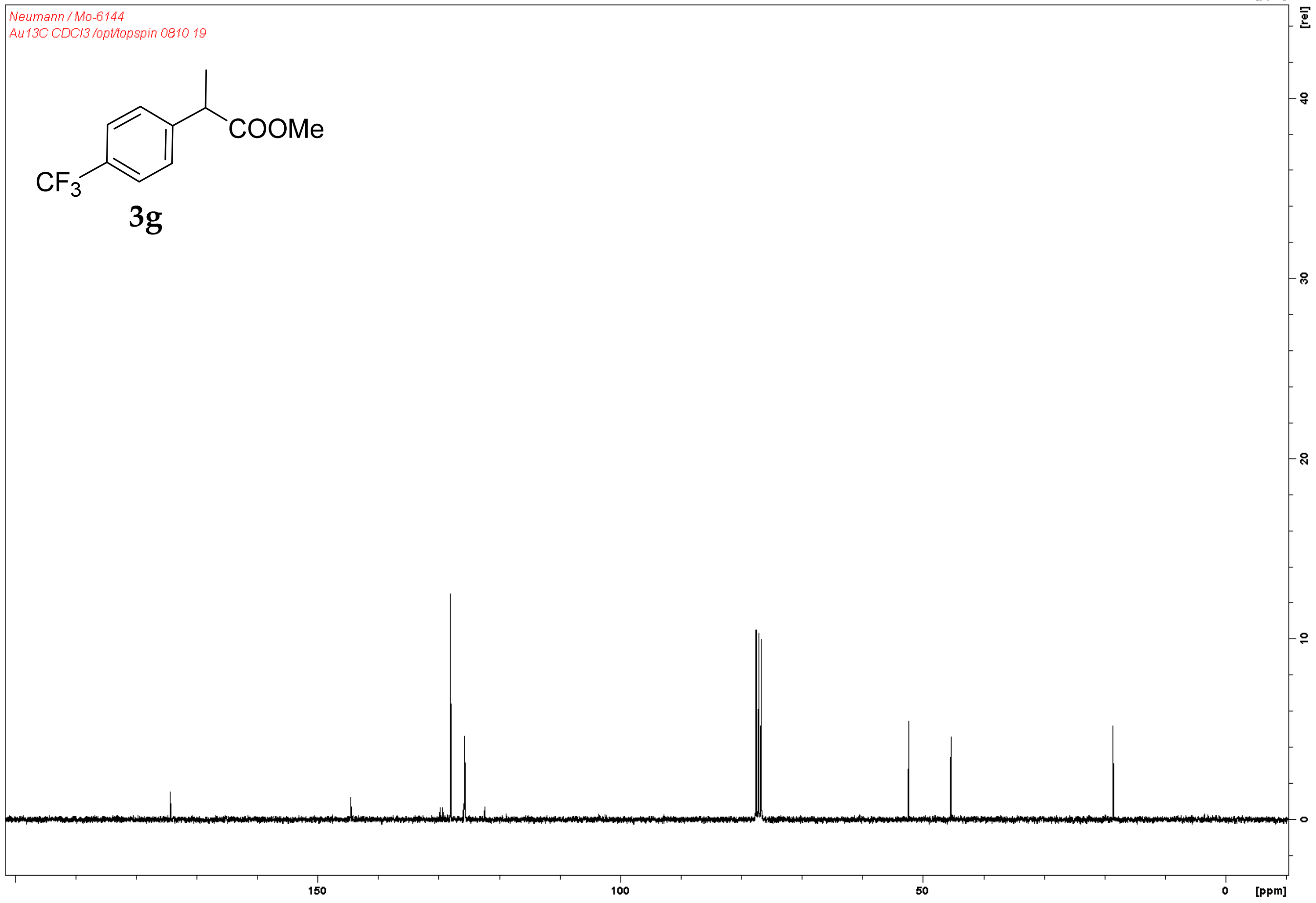
Neumann / Mo-6144
Au13C CDCl3 /opt/topspin 0810 19
COOMe
CF3
3g
[rel]
[ppm]

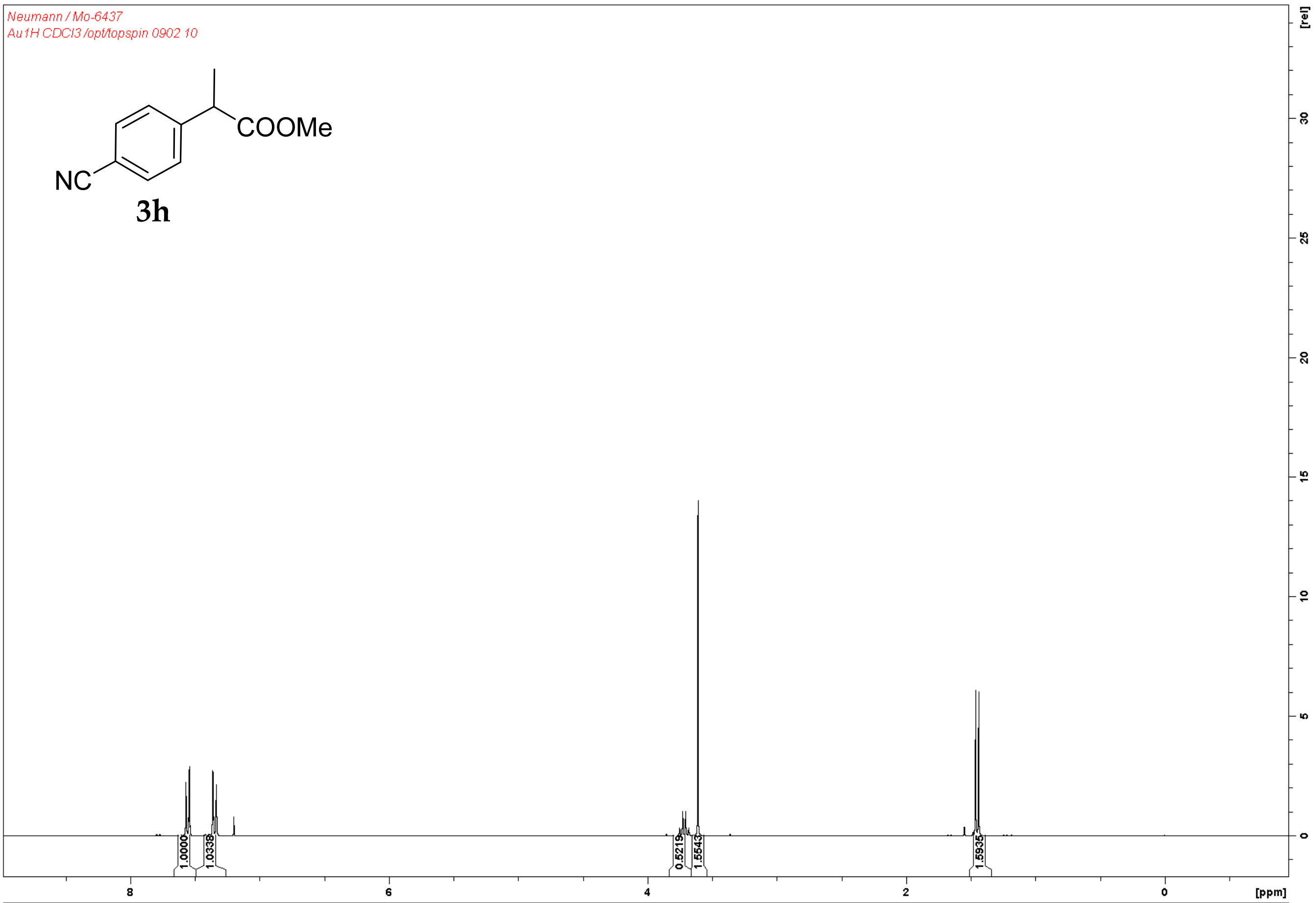

Neumann / Mo-6437
Au1H CDCl3 /opt/topspin 0902 10
COOMe
NC
3h
1.0000
1.0339
0.5219
1.5543
1.5935
[rel]
[ppm]

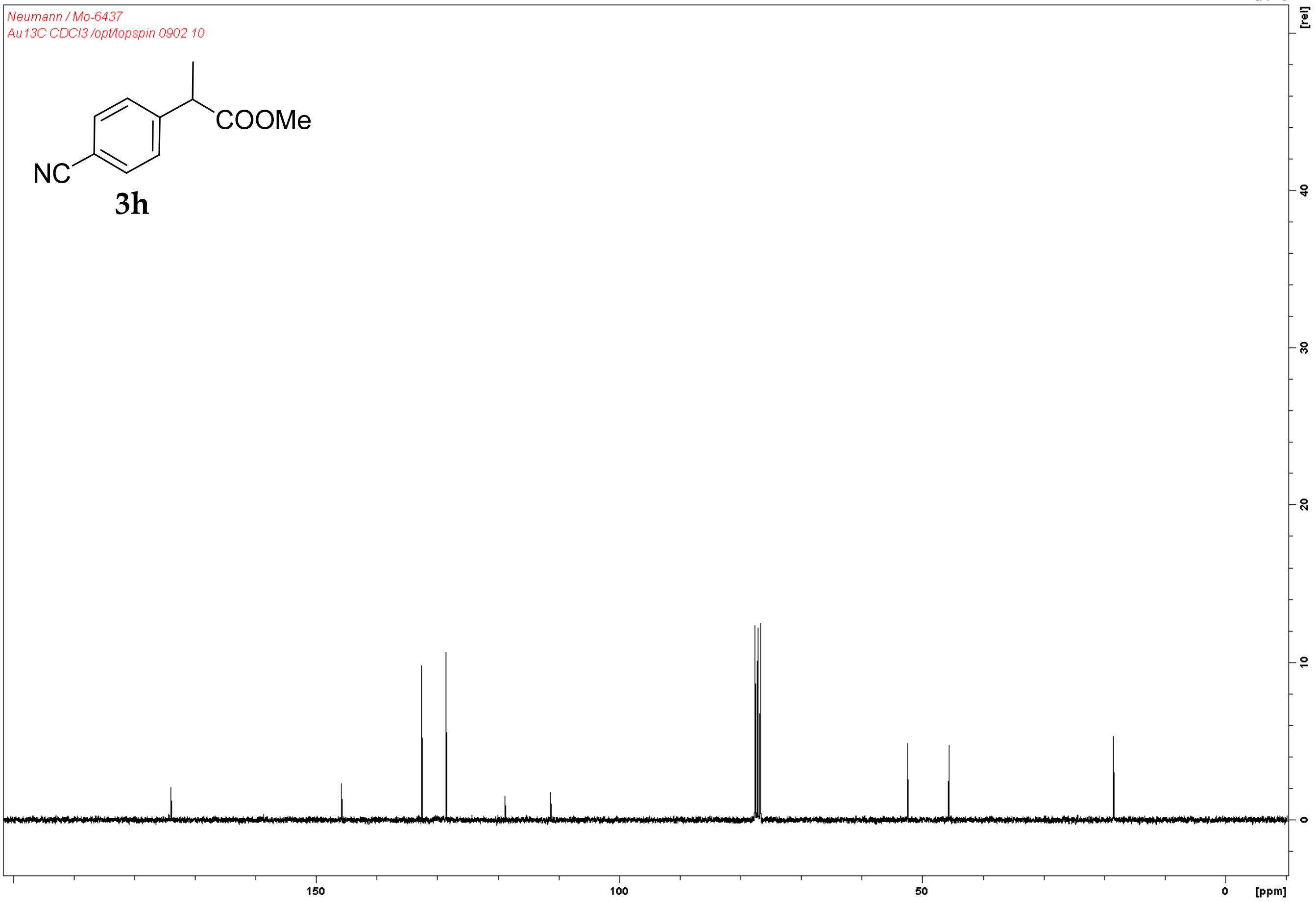

Neumann / Mo-6437
Au13C CDCl3 /opt/topspin 0902 10
COOMe
NC
3h
[rel]
[ppm]

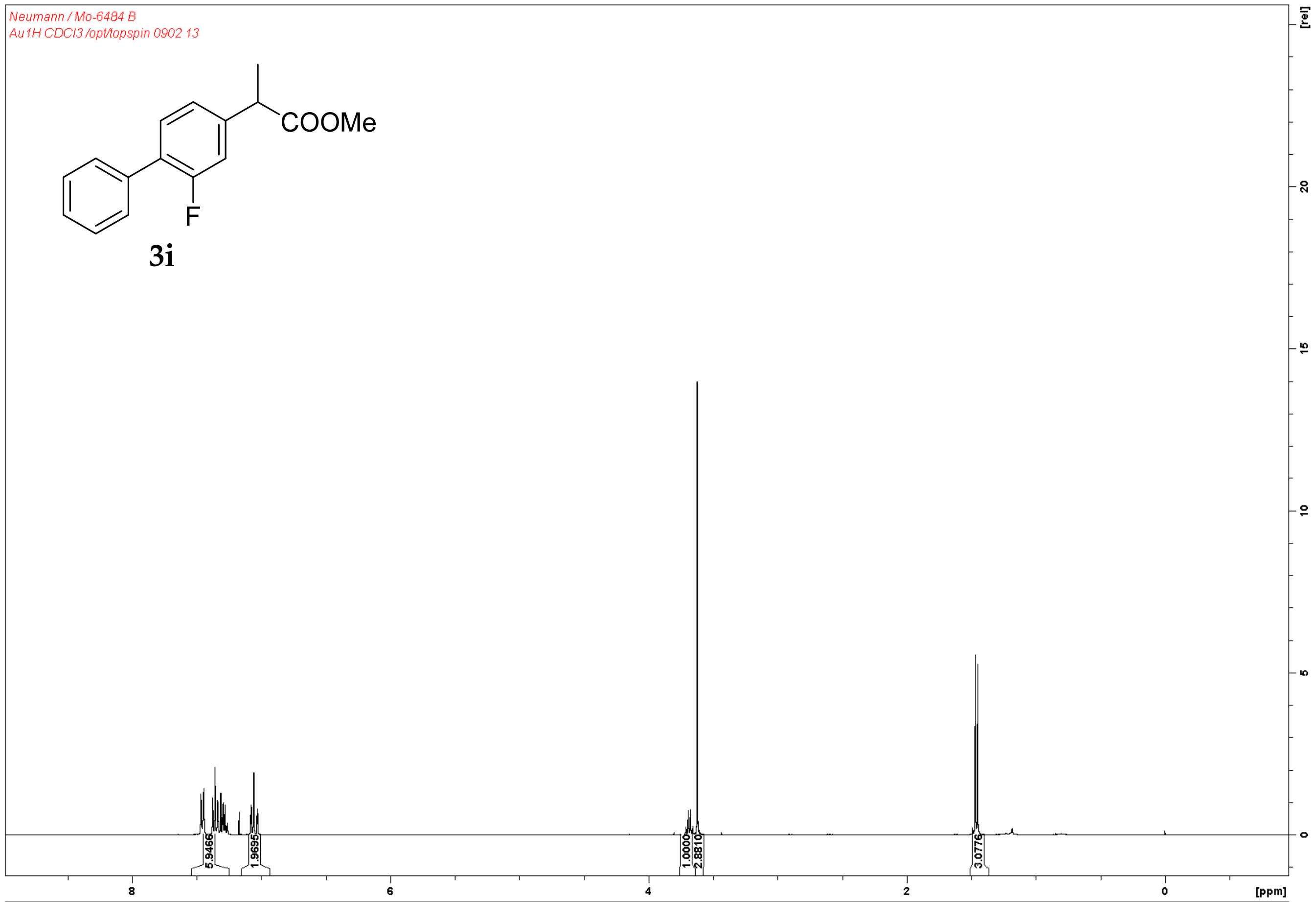
Neumann / Mo-6484 B
Au1H CDCl3 /opt/topspin 0902 13
COOMe
F
3i
5.9466
1.9695
1.0000
2.8810
3.0776
[rel]
[ppm]

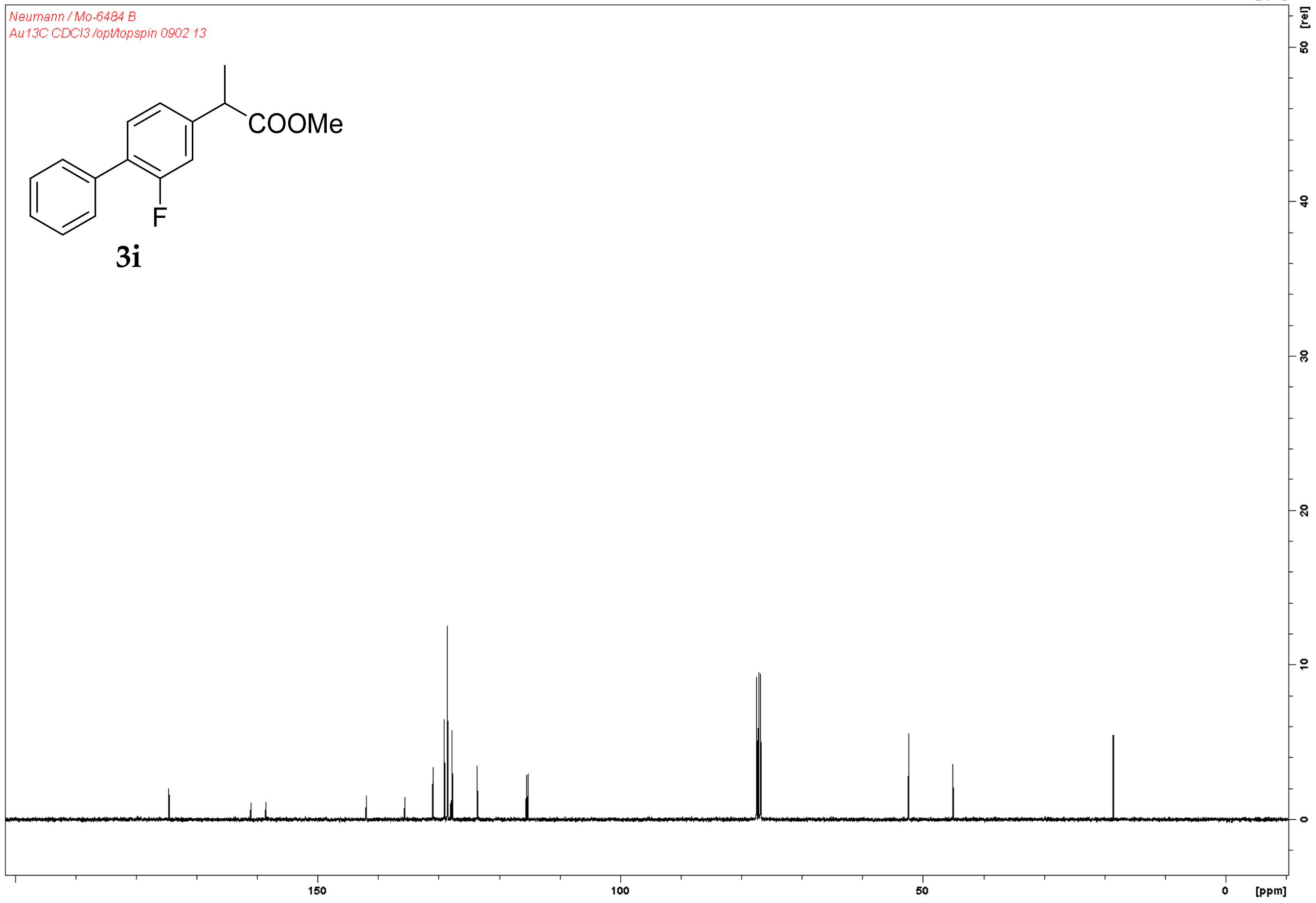
Neumann / Mo-6484 B
Au13C CDCl3 /opt/topspin 0902 13
COOMe
F
3i
[rel]
[ppm]

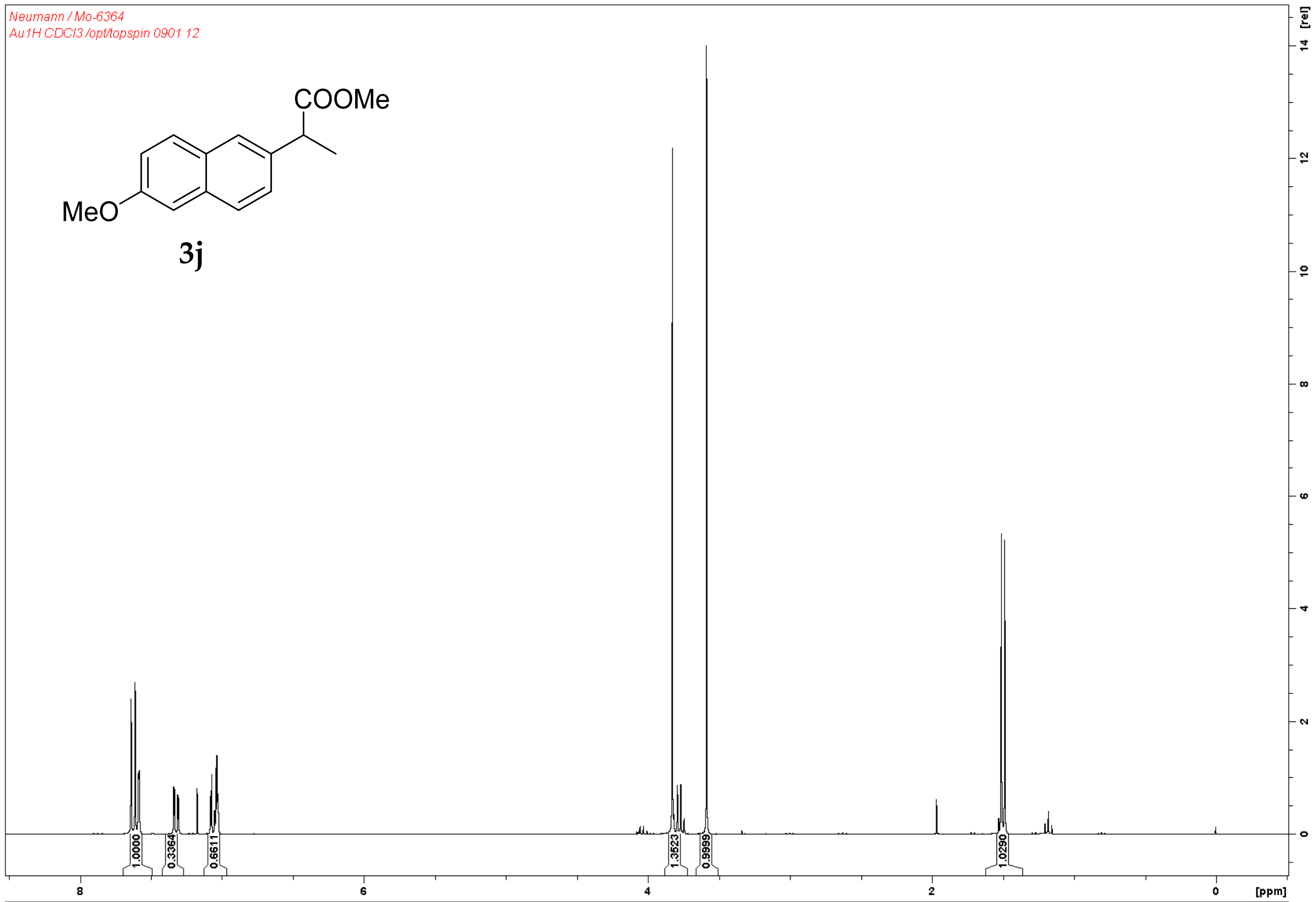
Neumann / Mo-6364
Au1H CDCl3 /opt/topspin 0901 12
COOMe
MeO
3j
1.0000
0.3364
0.6611
1.3523
0.9999
1.0290
[ppm]
[rel]

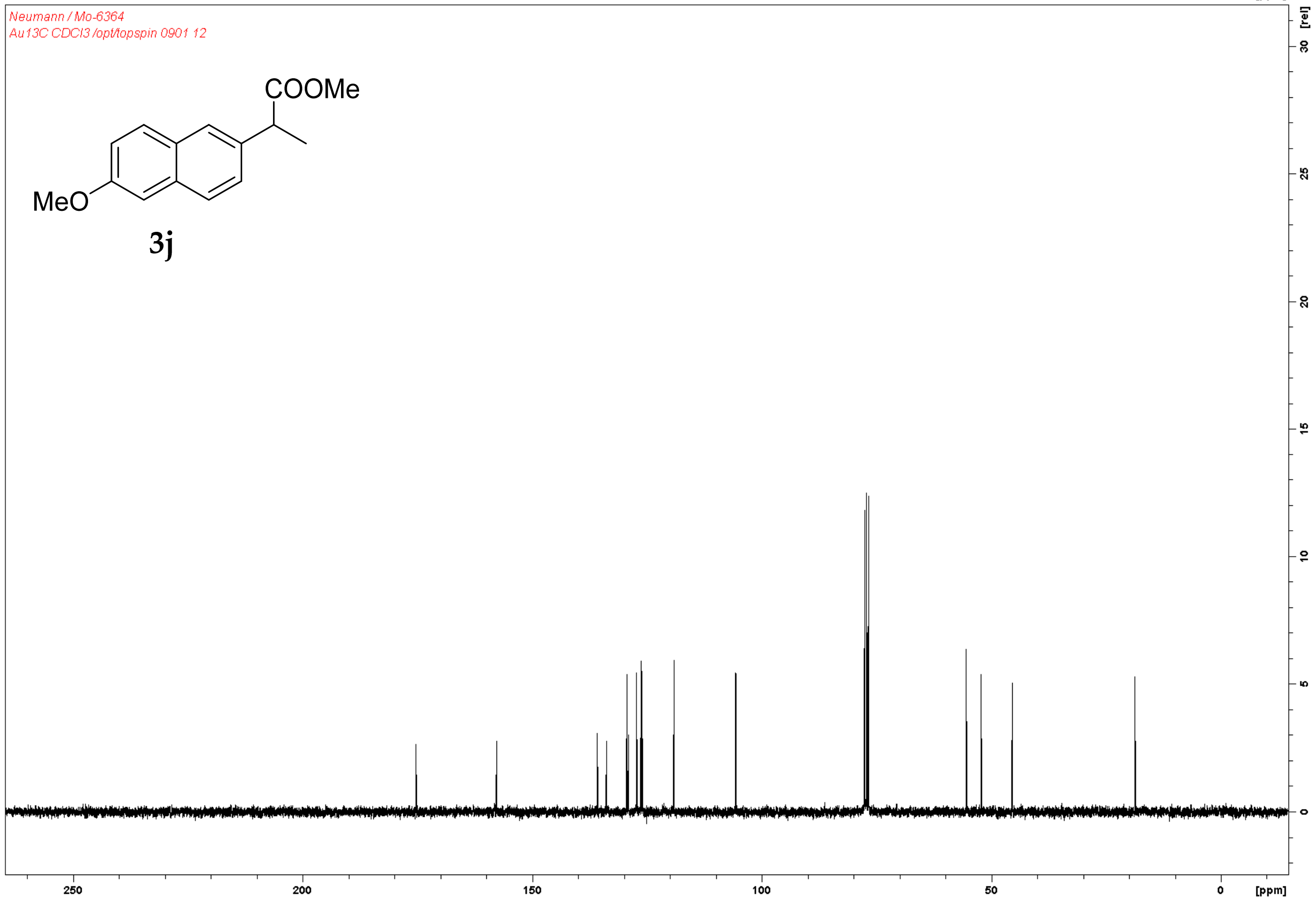
Neumann / Mo-6364
Au13C CDCl3 /opt/topspin 0901 12
COOMe
MeO
3j
[ppm]
[rel]

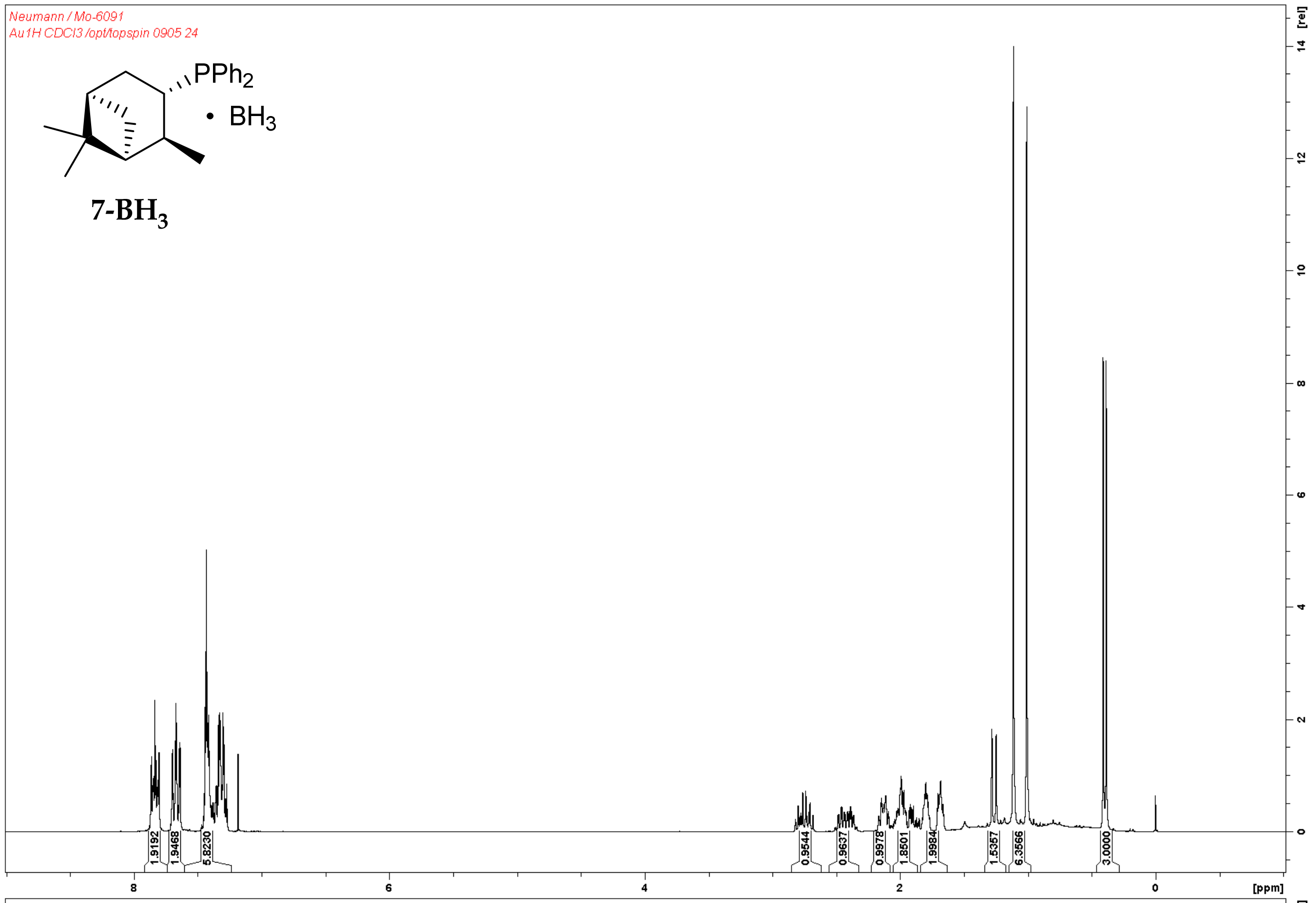
Neumann / Mo-6091
Au1H CDCl3 /opt/topspin 0905 24
PPh2
• BH3
7-BH3
[ppm]
[rel]

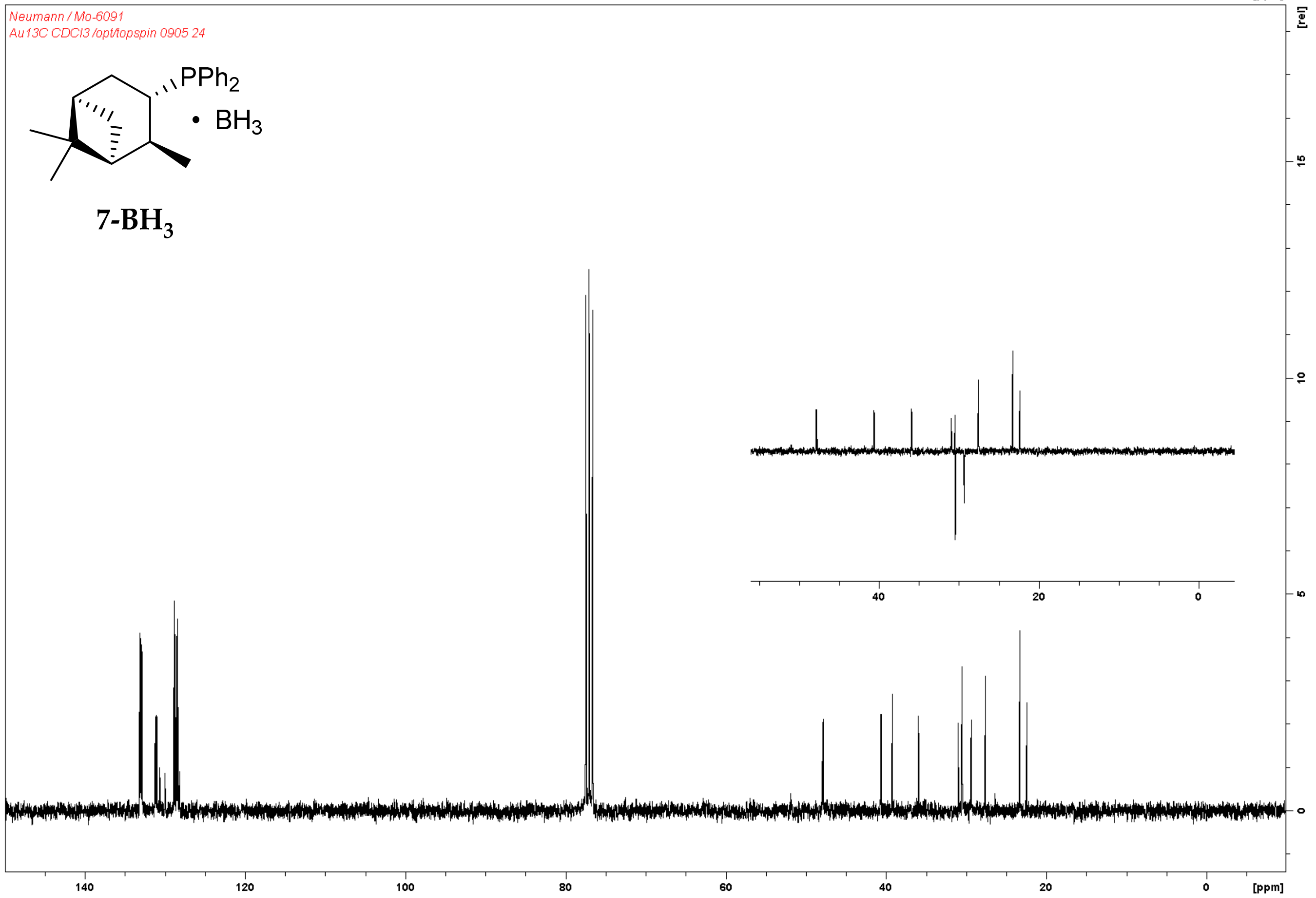
Neumann / Mo-6091
Au13C CDCl3 /opt/topspin 0905 24
PPh2
• BH3
7-BH3
[ppm]
[rel]

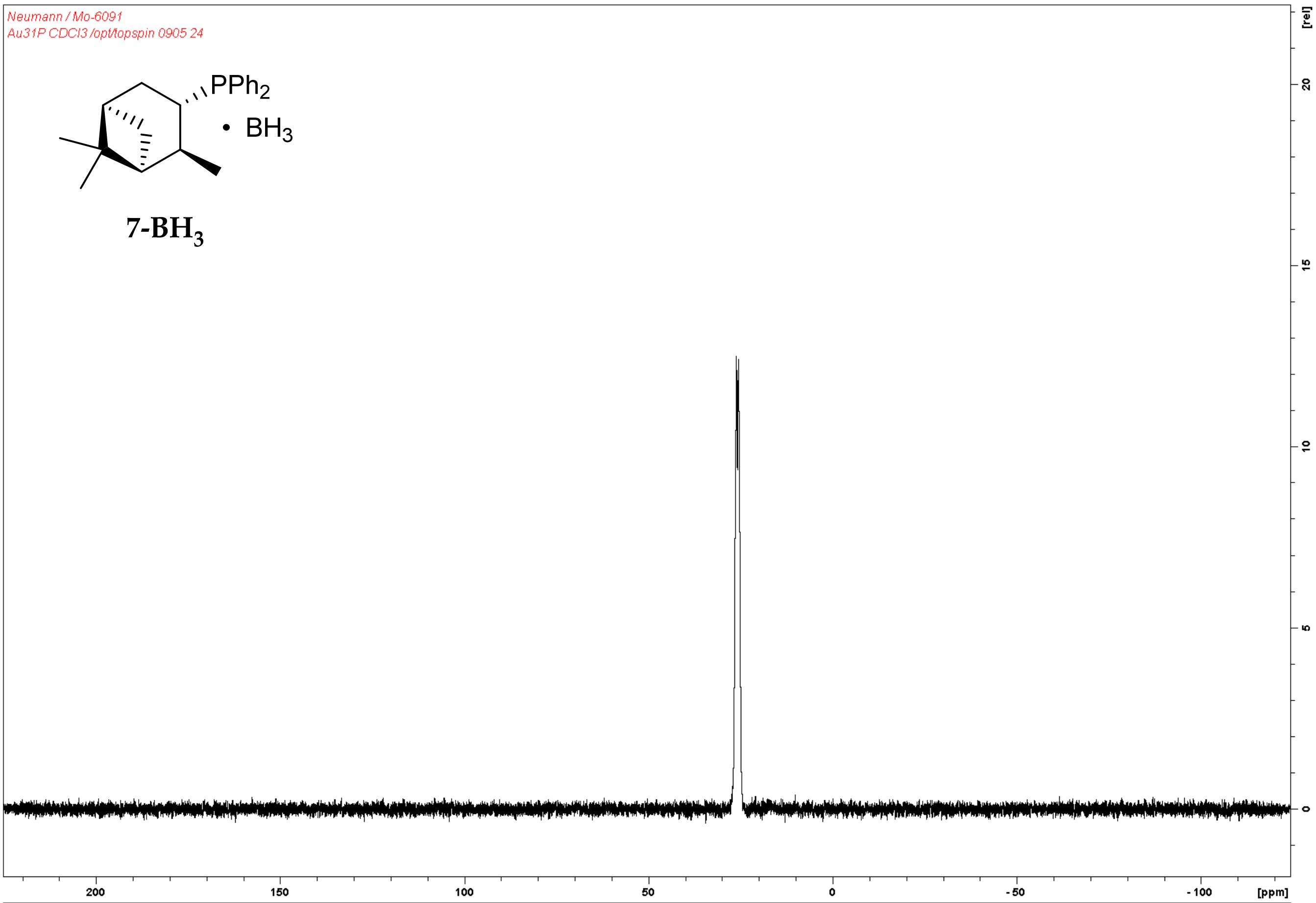
Neumann / Mo-6091
Au31P CDCl3 /opt/topspin 0905 24
PPh2
• BH3
7-BH3
[rel]
[ppm]

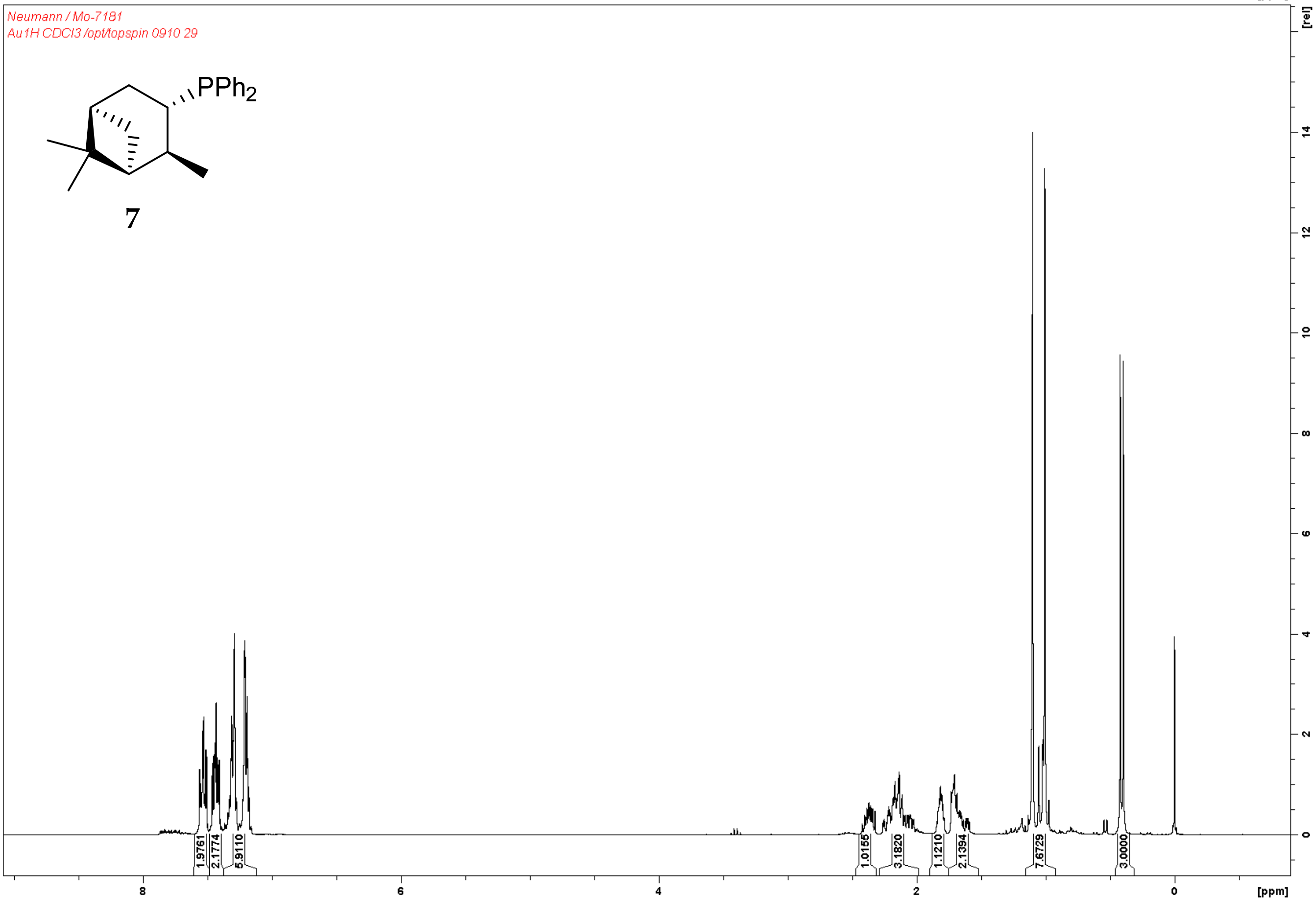
Neumann / Mo-7181
Au1H CDCl3 /opt/topspin 0910 29
PPh2
7
1.9761
2.1774
5.9110
1.0155
3.1820
1.1210
2.1394
7.6729
3.0000
[rel]
[ppm]

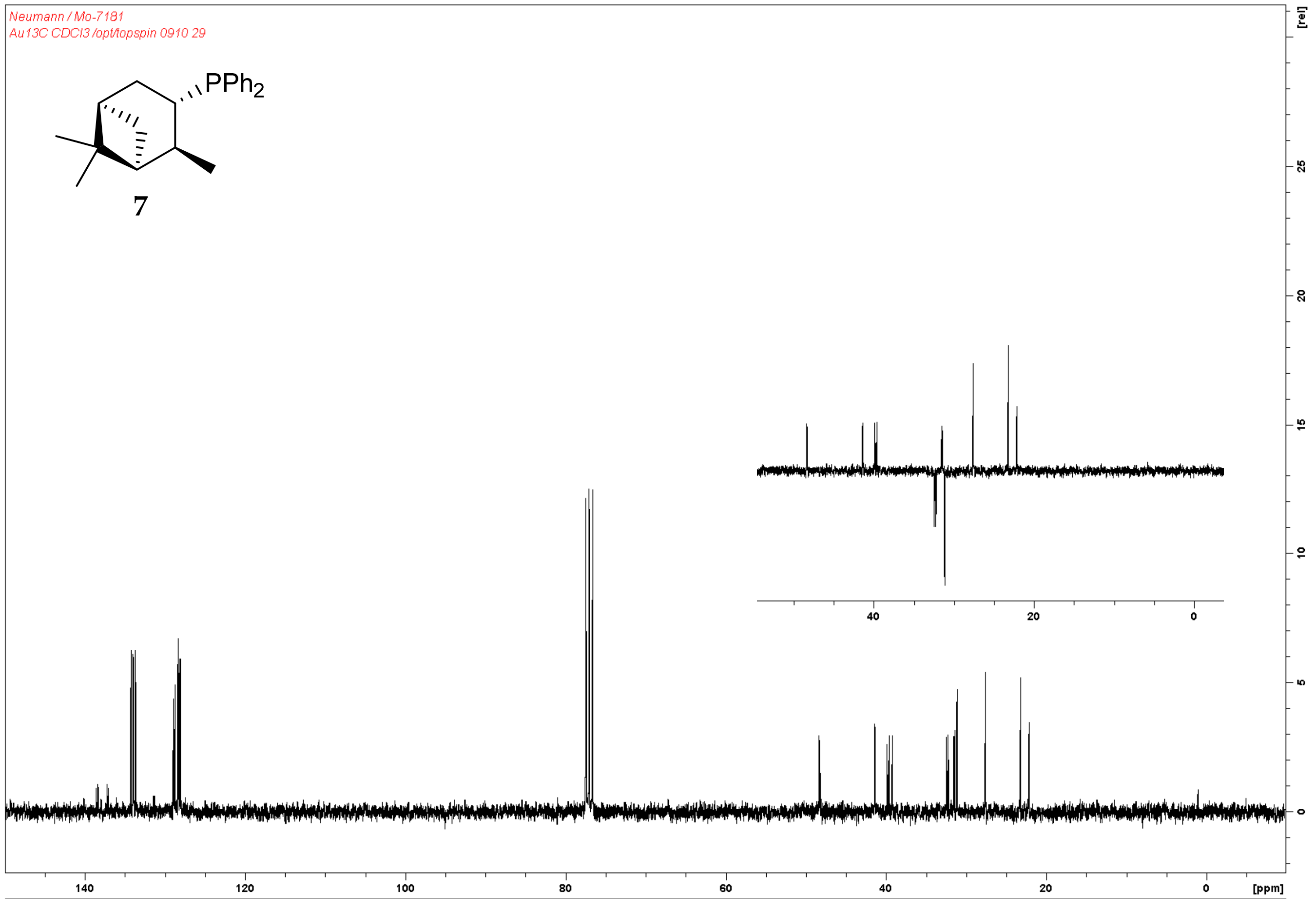
Neumann / Mo-7181
Au13C CDCl3 /opt/topspin 0910 29
PPh2
7
[rel]
[ppm]

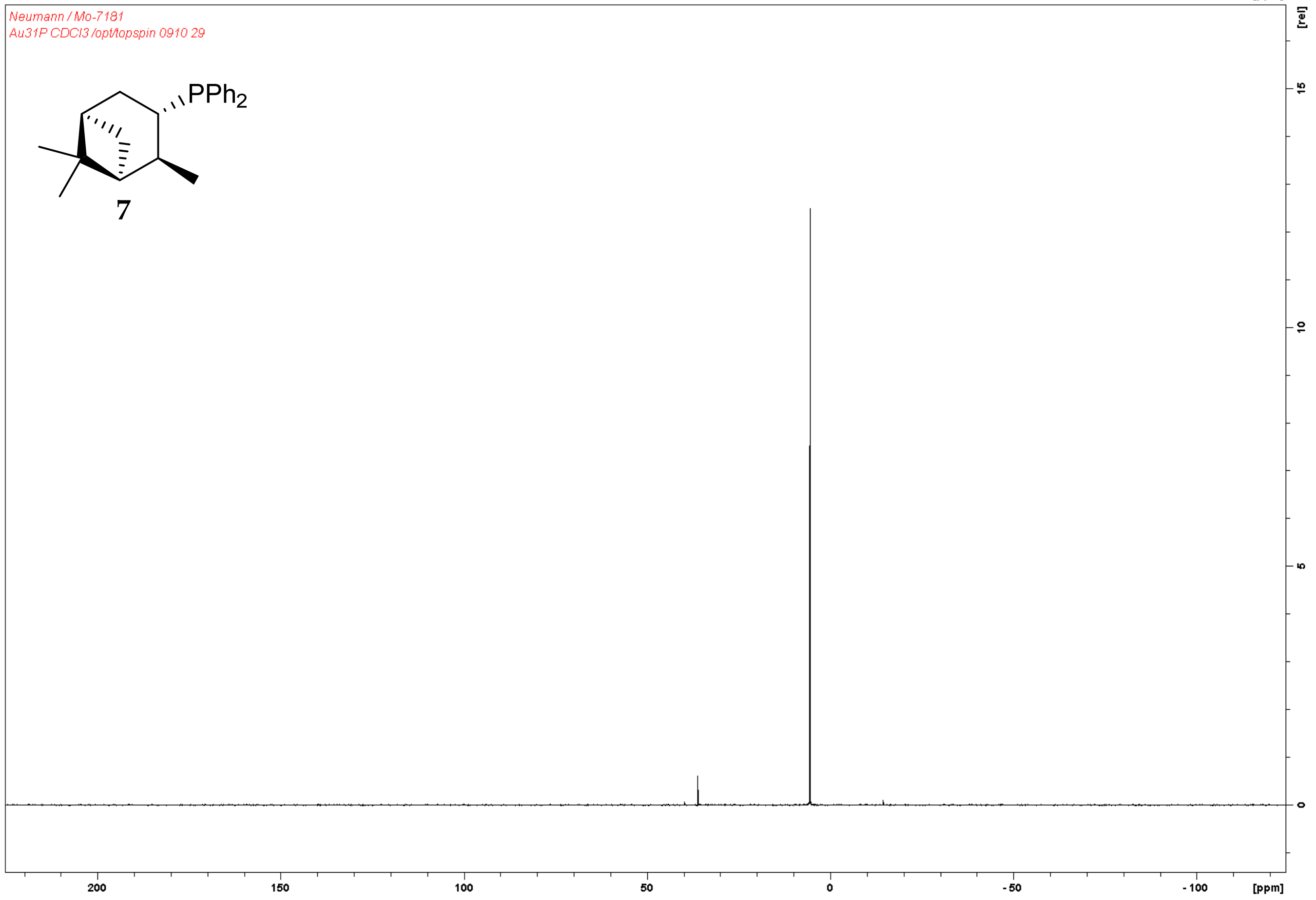
Neumann / Mo-7181
Au31P CDCl3 /opt/topspin 0910 29
PPh2
7
[rel]
[ppm]

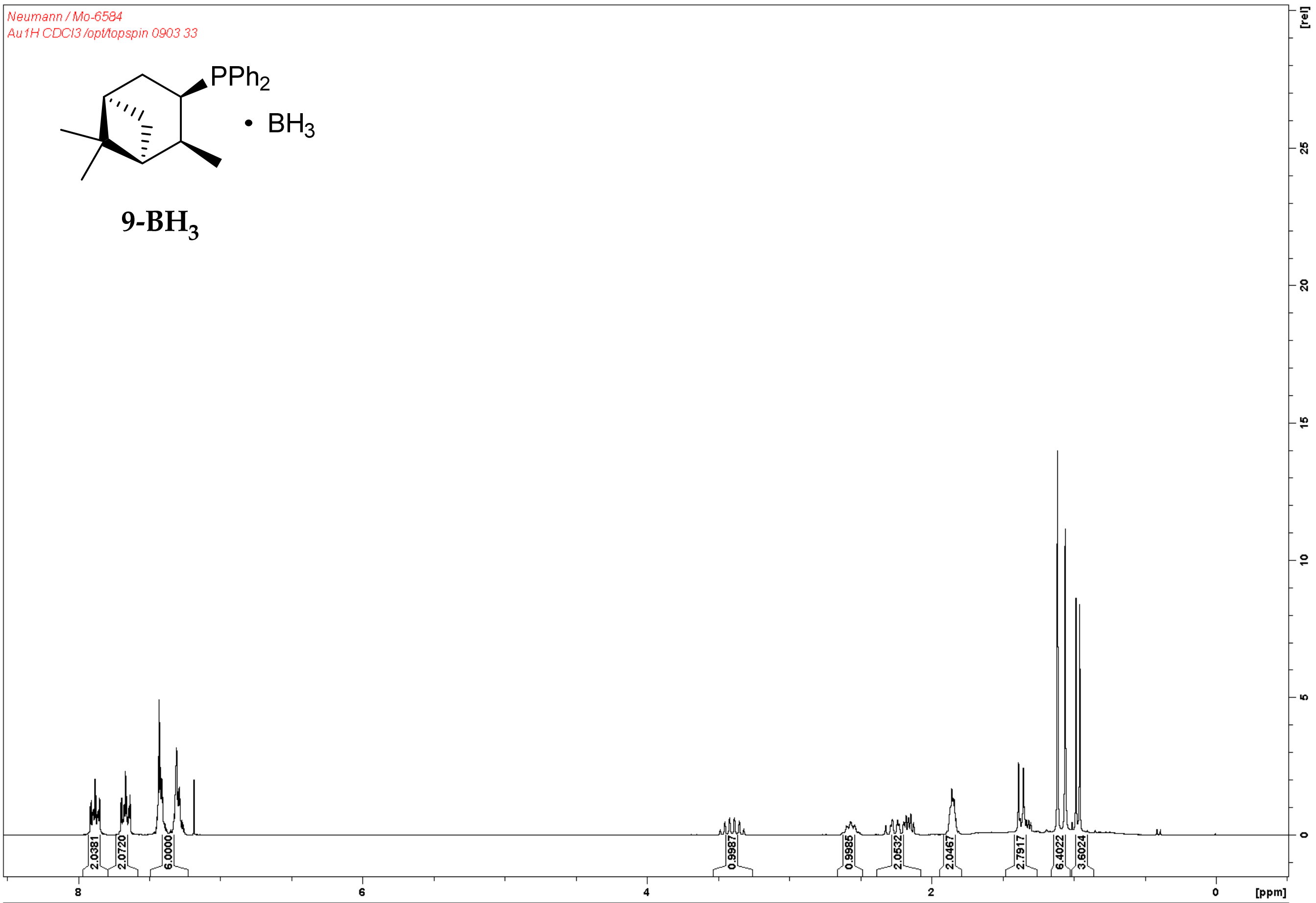
Neumann / Mo-6584
Au1H CDCl3 /opt/topspin 0903 33
PPh2
• BH3
9-BH3
2.0381
2.0720
6.0000
0.9987
0.9985
2.0532
2.0467
2.7917
6.4022
3.6024
8
6
4
2
0
[ppm]
[rel]

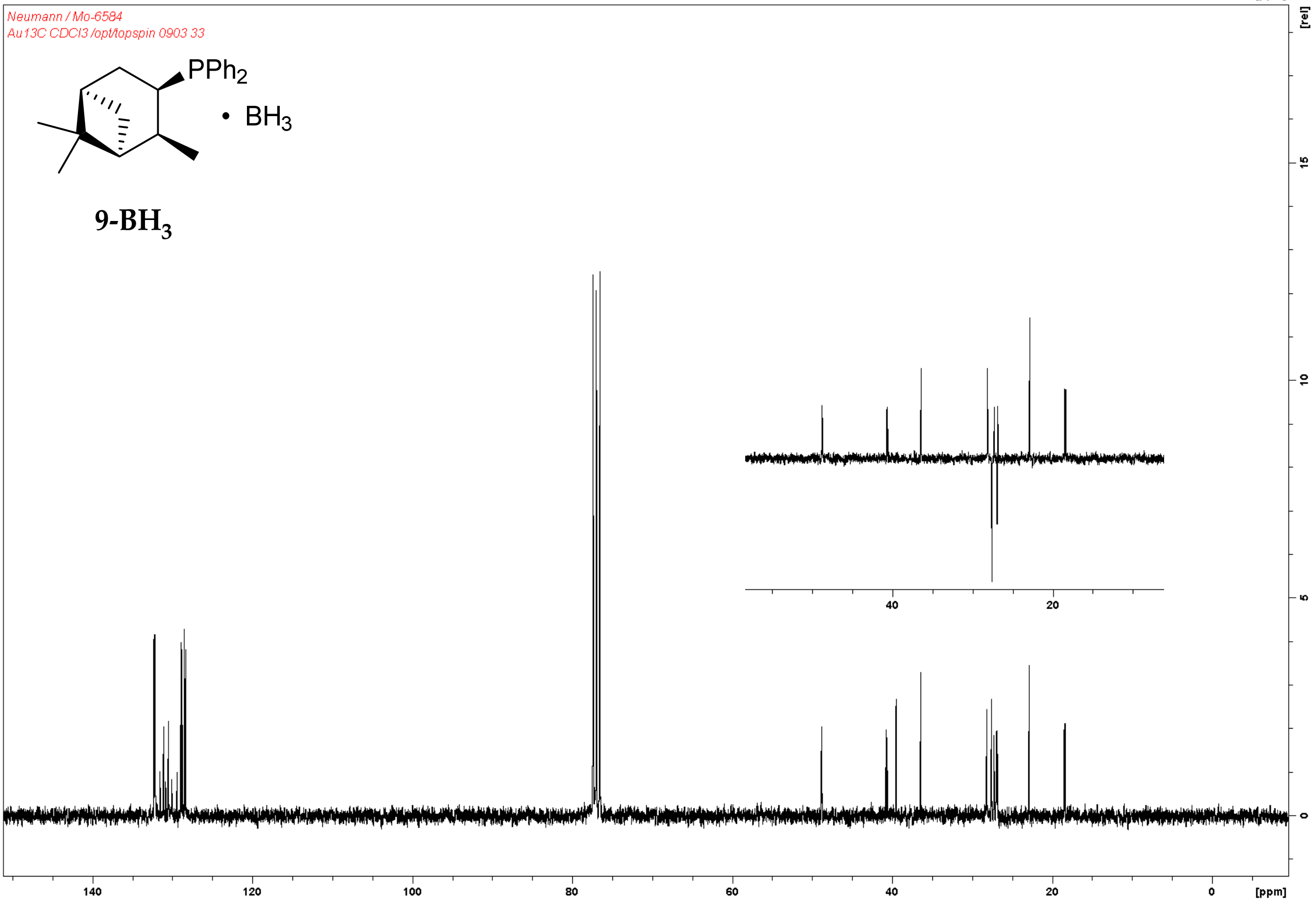
Neumann / Mo-6584
Au13C CDCl3 /opt/topspin 0903 33
PPh2
• BH3
9-BH3
40
20
140
120
100
80
60
40
20
0
[ppm]
[rel]

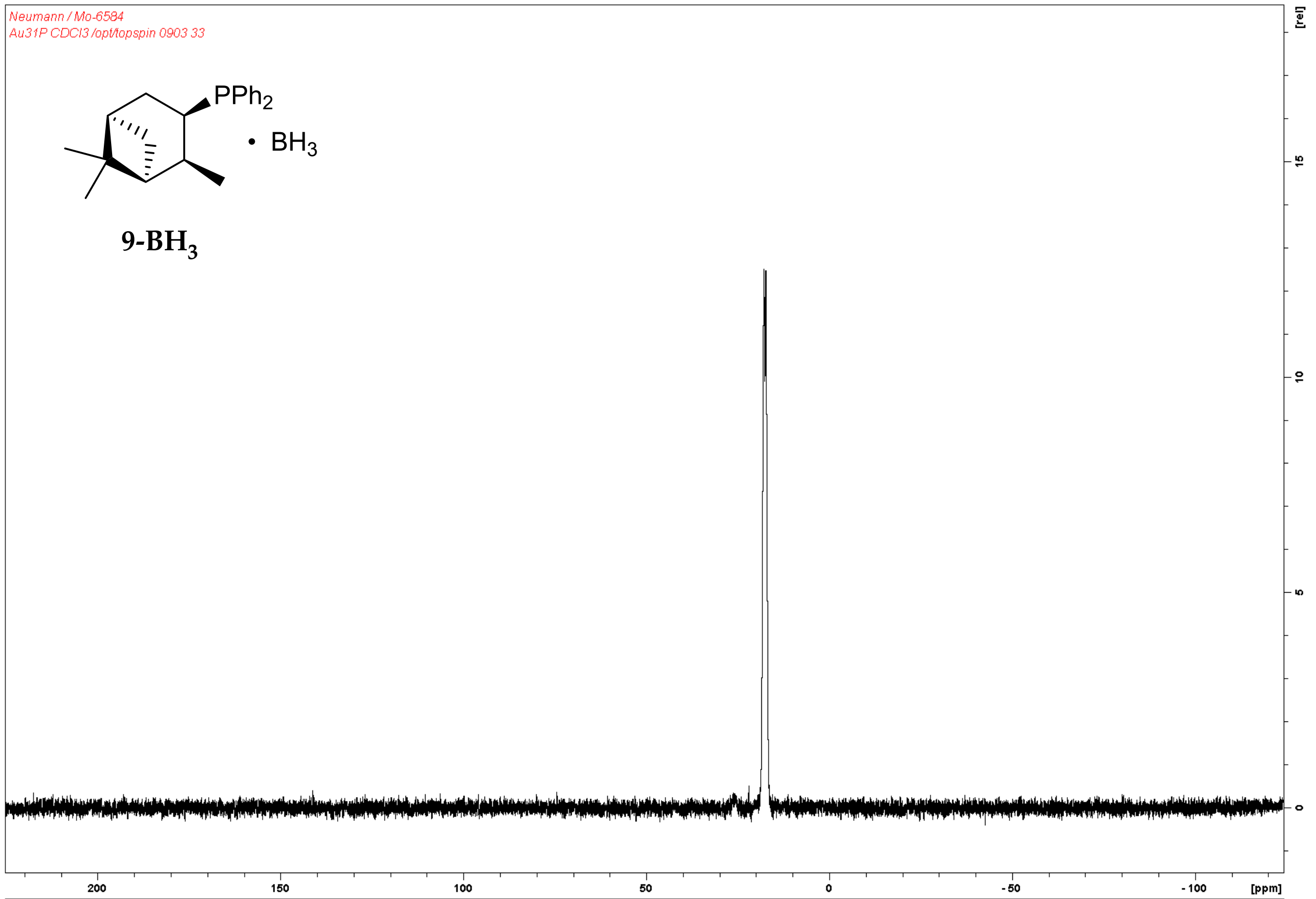
Neumann / Mo-6584
Au31P CDCl3 /opt/topspin 0903 33
PPh2
• BH3
9-BH3
[rel]
[ppm]

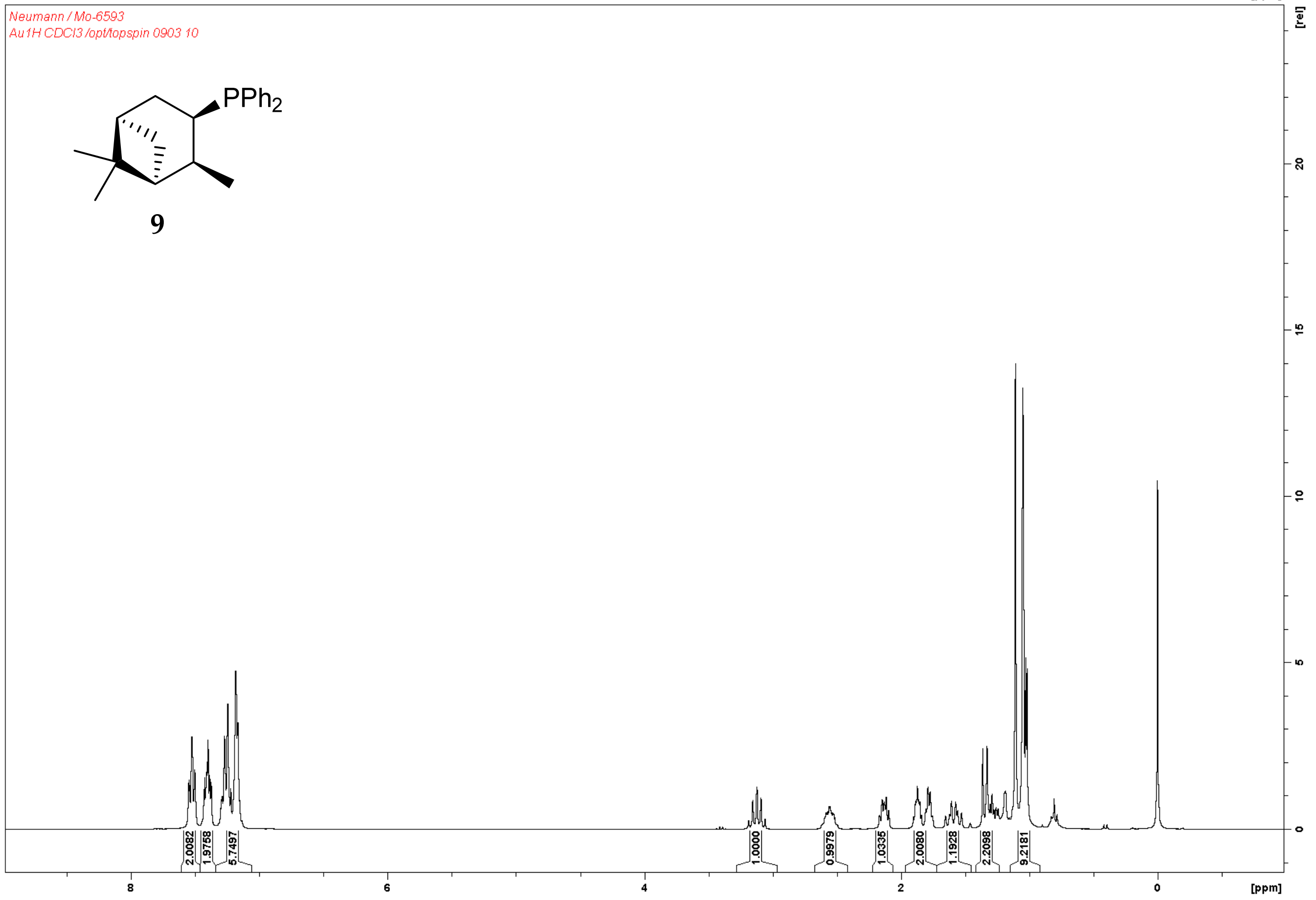
Neumann / Mo-6593
Au1H CDCl3 /opt/topspin 0903 10
PPh2
9
2.0082
1.9758
5.7497
1.0000
0.9979
1.0335
2.0080
1.1928
2.2098
9.2181
[rel]
[ppm]

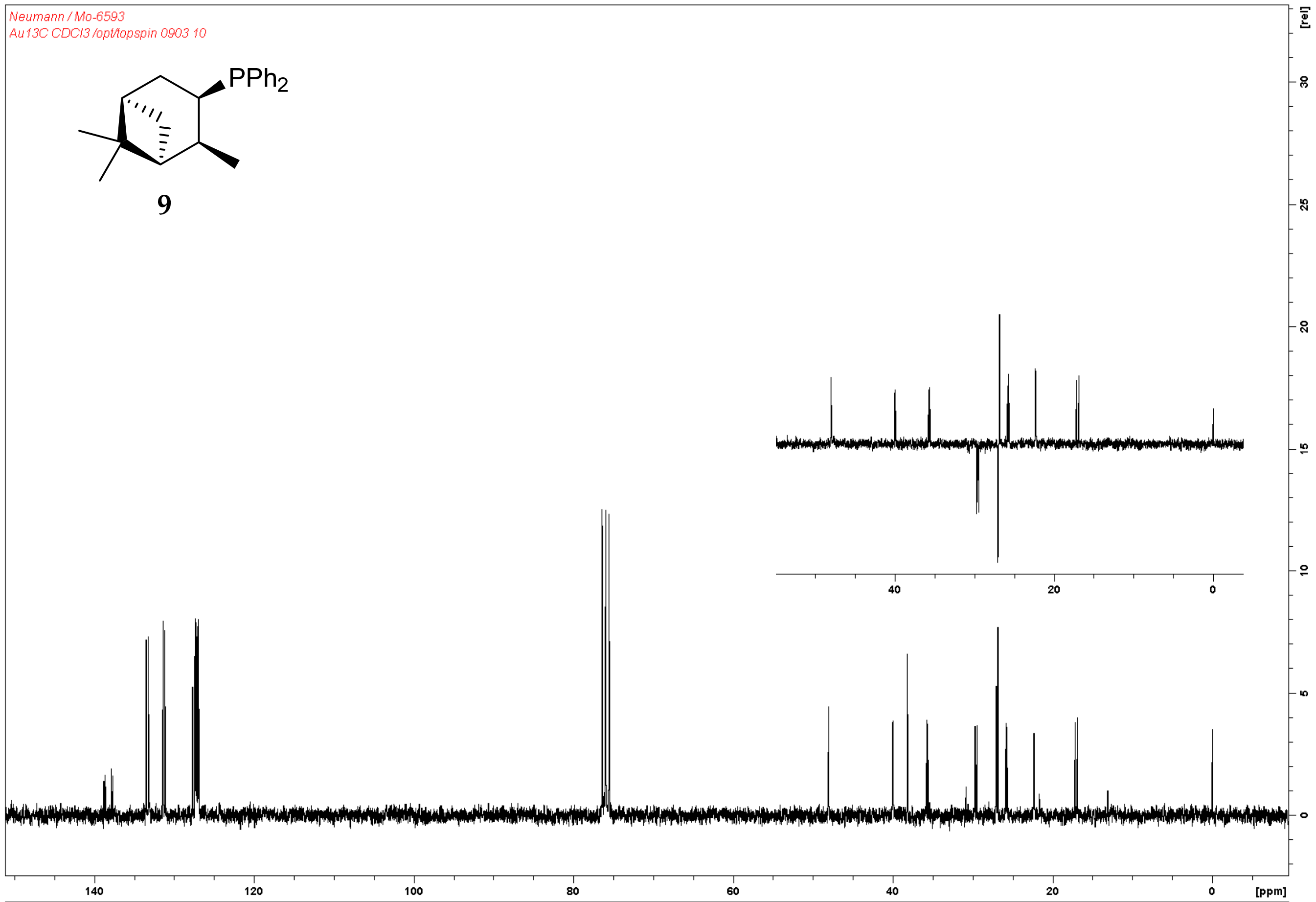
Neumann / Mo-6593
Au13C CDCl3 /opt/topspin 0903 10
PPh2
9
[rel]
[ppm]

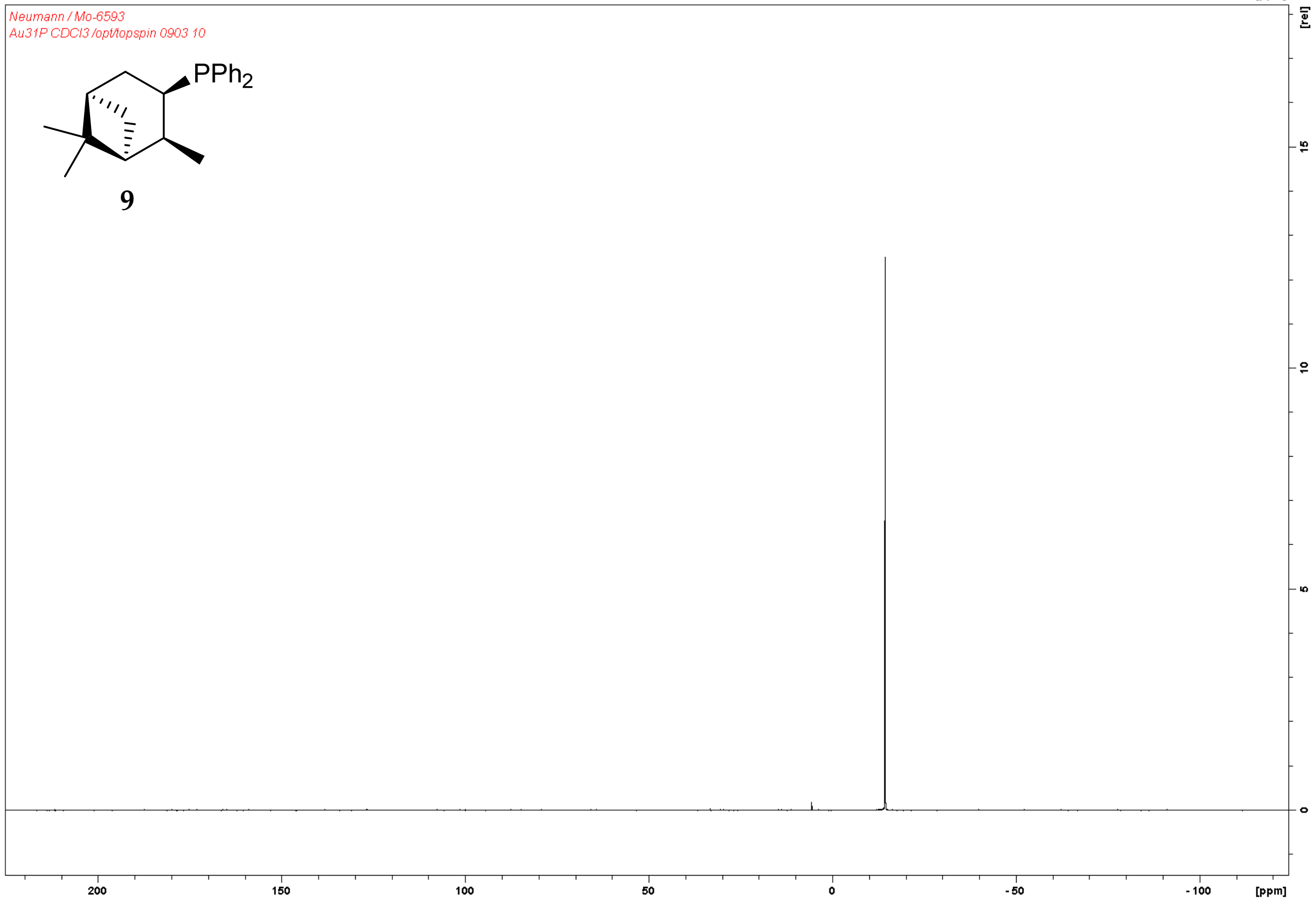
Neumann / Mo-6593
Au31P CDCl3 /opt/topspin 0903 10
PPh2
9
[rel]
[ppm]
